# A principled approach to non-discrimination in cost-effectiveness

**DOI:** 10.1007/s10198-023-01659-7

**Published:** 2024-02-27

**Authors:** Darius N. Lakdawalla, Jason N. Doctor

**Affiliations:** Schaeffer Center for Health Policy and Economics, University of Southern California, Los Angeles, USA

**Keywords:** Cost-effectiveness, Equity, Equal value of life-years gained, Health years in total, Generalized risk-adjusted cost-effectiveness, I11, I13, I14, I18

## Abstract

The US Inflation Reduction Act (IRA) prohibits the Centers for Medicare and Medicaid Services (CMS) from using standard quality-adjusted life-years or other value assessment methods that discriminate against the aged, terminally ill, or disabled when setting maximum fair prices for prescription drugs. This policy has reignited interest in methods for assessing value without discrimination. Equal value of life-years gained (EVL), healthy years in total (HYT), and Generalized Risk-Adjusted Cost-Effectiveness (GRACE) have emerged as proposals. Neither EVL nor HYT rests on well-articulated microeconomic foundations. We show that they produce decisions that are inconsistent over time in a variety of ways, including: (1) failure to support additivity and indirect comparison in cases where the standard-of-care therapy changes over time; (2) strictly negative value of survival gains that accrue from a new, better standard-of-care, particularly for the disabled themselves; (3) unbounded average value of survival gains; and (4) non-convex survival preferences. We propose an alternative method that relies on GRACE and its microeconomic foundations.

## Introduction

The US Inflation Reduction Act (IRA) directed the Centers for Medicare and Medicaid Services (CMS) to negotiate “maximum fair prices” (MFPs) for certain prescription medicines. CMS must consider a range of factors in this determination, including comparative effectiveness, unmet need, scientific novelty, and costs of comparators. However, echoing similar language from the Affordable Care Act (ACA) [1], the IRA statute explicitly forbids CMS from using any evidence or method “in a manner that treats extending the life of an individual who is elderly, disabled, or terminally ill as of lower value than extending the life of an individual who is younger, nondisabled, or not terminally ill” [2, p. 36]. This US legislative development has renewed interest in value assessment methods that avoid discrimination against individuals with illness or disability, including Equal Value of Life-years gained (EVL) [3], Health Years in Total (HYT) [4], and Generalized Risk-Adjusted Cost-Effectiveness (GRACE) [1].

Conventional cost-effectiveness analysis (CEA) based on quality-adjusted life-years (QALYs) implies that life-extension is always less valuable in lower quality-of-life states. This implication conflicts with empirical evidence: consumers appear to place more value on improvements for the severely ill [5, 6] as do third-party payers [7, 8]. In response, Nord et al. [3] proposed the concept of “cost-value analysis,” which weighted gains in health-related utility by the severity of the condition being treated and by the consumer’s capacity to benefit. Cost-value analysis allows the analyst to place more weight on health improvements for consumers with more limited potential to benefit, e.g., the disabled.

Shortly after Nord et al.’s proposal, however, Østerdal [9] demonstrated that cost-value analysis leads to counter-intuitive outcomes inconsistent with a social welfare function. Nonetheless, one aspect of the Nord et al.’s proposal retained an enduring influence, namely, the principle that life-extension should be equally valuable to all, regardless of whom it accrues to. This equal value of life-years (EVL) principle continues to be employed by the Institute for Clinical and Economic Review in the US [10, 11] and to be considered for use in US federal government decisions around drug pricing.

While EVL may appear intuitive, it also violates several other widely held and intuitive principles. Hasman and Østerdal [12] show that any social welfare ordering satisfying the EVL principle will violate both the weak and the strong forms of the Pareto principle. Thus, an ordering based on EVL can lead to a paternalistic outcome where every consumer in society prefers Policy A to Policy B, but the social welfare ordering prioritizes Policy B. Fleurbaey and Ponthière [13] demonstrate that the EVL principle is inconsistent with the strict social desirability of quality-of-life improvements or with prioritizing life-extension for the disadvantaged. Moreno-Ternero and Østerdal [14] demonstrate that the strong EVL principle (where life-extension is equally valuable in all settings), coupled with standard axioms of social preference, leads to a social welfare function that maximizes life expectancy without regard to quality-of-life. Quality-of-life preferences re-emerge only if one narrows the scope of EVL to assure equal value of life-extension to consumers with the same quality-of-life level; this narrower version of EVL, however, is likely to violate the anti-discrimination provisions in the US Inflation Reduction Act (IRA) and Affordable Care Act (ACA).

Basu et al. proposed the HYT metric to address the inconsistency between EVL and preferences for quality-of-life [4]. HYT adds the correlation between survival gains and quality-of-life gains to the EVL metric [4]; therefore, HYT values quality-of-life improvements that coincide with the addition of life-years. However, just like cost-value analysis, neither EVL nor HYT follow from principled and axiomatic economic theory. Thus, while both metrics address real discordance between theory and consumer preferences, they each introduce ad hoc adjustments that leave them vulnerable to logical inconsistencies and/or counter-intuitive implications [15, 16].

This paper aims to inform the US health policy discussion by studying the three specific metrics of incremental value under consideration by CMS: the EVL metric, as formulated by ICER [16], the HYT metric [4], and GRACE [1, 17–20]. We present a focused set of intuitive principles sufficient to reveal how ICER’s EVL metric and the HYT metric violate commonly held assumptions, including: additivity and indirect comparison; monotonicity and convexity in preferences for survival; and boundedness in the average value of survival. We also demonstrate circumstances under which these two metrics can discriminate against sicker and disabled populations, the very outcome they were engineered to avoid. Finally, we offer an alternative approach to non-discriminatory cost-effectiveness analysis using GRACE, which proceeds from a well-defined consumer utility framework and thus readily satisfies standard economic axioms. And, while GRACE does not universally avoid discrimination against the sick and disabled, plausible conditions exist that ensure non-discrimination [1]. We identify and enumerate these conditions to offer a strategy for principled, non-discriminatory value assessment. Notably, we do not attempt to provide characterization results for any of these metrics. In the first place, it is unclear that the ICER EVL and HYT metrics can be derived from plausible axioms. Moreover, GRACE is simply a generalized form of traditional cost-effectiveness analysis, so that characterization results are less novel and useful. Instead, we aim to compare and contrast three approaches that remain under active consideration for use in the US context.

Sect. "Theoretical preliminaries" begins by setting forth a set of focused principles for evaluating the validity and usefulness of non-discriminatory metrics of value. Sect. "Principle violations within the EVL and HYT metrics" characterizes the decision-making pathologies that arise in ICER’s EVL and HYT. Sect. "A consistent choice-theoretic approach to eliminating discrimination" explains how GRACE comports with the same principles of value assessment and how it can be used to eliminate discrimination against the sick and disabled. Sect. "Conclusion" concludes.

## Theoretical preliminaries

We begin by summarizing the EVL,^1^ HYT, and GRACE decision metrics, along with a focused set of plausibly uncontroversial principles for non-discriminatory value assessment.

## Summary of decision metrics

Consider a standard-of-care therapy, $X$, and a novel technology $Y$. These are characterized by sequences of survival probabilities and quality-of-life weights, $\left\{ {S_{Xt} ;Q_{Xt} } \right\}_{t = 1}^{T}$ and $\left\{ {S_{Yt} ;Q_{Yt} } \right\}_{t = 1}^{T}$. For instance, $S_{Xt}$ is the probability of surviving from time $1$ to time $t$, and $Q_{Xt}$ is the quality-of-life weight at time $t$, when treated by intervention $X$. Incremental quality-adjusted life-years (QALYs) for $Y$ compared to $X$ are given by

1 $$\Delta {\text{QALY}}\left( {Y,X} \right) \equiv \mathop \sum \limits_{t} \left( {S_{Yt} Q_{Yt} - S_{Xt} Q_{Xt} } \right) = \mathop \sum \limits_{t} \left( {S_{Yt} \left( {Q_{Yt} - Q_{Xt} } \right) + \left( {S_{Yt} - S_{Xt} } \right)Q_{Xt} } \right).$$

Notice that survival gains are worth less to patients with lower initial quality-of-life, a property of the QALY that has received criticism [3].

In response to this criticism, EVL calculates the total gain in life-years and then adds the expected gain in quality-of-life using survival probabilities for the standard-of-care technology with the lowest total survival. Notice that the latter technology may not be part of the pairwise comparison, causing EVL to violate the independence of irrelevant alternatives [15].

Define $\chi$ as the set of all standard-of-care technologies. Define $S\left( X \right) \equiv \sum\nolimits_{t} {S_{Xt} }$ as the “total survival” of a given standard-of-care technology $X \in \chi$, and suppose $X^{*} \in \chi$ is the technology in $\chi$ with the lowest total survival. Incremental EVL for the novel technology $Y$ can be formally defined as [4, 16, 21]

2 $$\Delta EVL\left( {Y,X^{*} } \right) = \left\{ {\begin{array}{*{20}c} {\mathop \sum \limits_{t} \left( {S_{Yt} - S_{{X^{*} t}} } \right) + \mathop \sum \limits_{t} S_{{X^{*} t}} \left( {Q_{Yt} - Q_{Xt} } \right),} & { {\text{if}} \mathop \sum \limits_{t} S_{{X^{*} t}} < \mathop \sum \limits_{t} S_{Yt} } \\ {\mathop \sum \limits_{t} \left\{ {S_{Yt} Q_{Yt} - S_{{X^{*} t}} Q_{{X^{*} t}} } \right\}, } & {{\text{if}} \mathop \sum \limits_{t} S_{{X^{*} t}} \ge \mathop \sum \limits_{t} S_{Yt} } \\ \end{array} } \right..$$

If the novel technology extends life over the standard-of-care, $\Delta{\text{EVL}} = { }\mathop \sum \limits_{t} \left( {S_{Yt} - S_{{X^{*} t}} } \right) + \mathop \sum \limits_{t} S_{{X^{*} t}} \left( {Q_{Yt} - Q_{Xt} } \right)$, total life-years gained, plus expected quality-of-life gains using the survival profile of the survival-minimizing standard of care. If the novel technology fails to extend life over $X^{*}$, the incremental EVL is simply the incremental QALY.^2^

$\Delta{\text{EVL}}$ has been criticized, because it disregards the value of quality-of-life improvements [4, 13, 14]. To excise this property, Basu, Carlson, and Veenstra have proposed incremental Health Years in Total (HYT). Unlike EVL, HYT uses the survival profile of the novel life-extending therapy—or, more generally, the therapy with the maximum total survival—when calculating the expected value of quality-of-life gains. As a result, HYT incorporates quality-of-life improvements that accrue during the period of life-extension. Suppose $\overline{X} \in \chi$ maximizes total survival over the set of standard-of-care technologies, $\chi$. Like ICER’s EVL metric, HYT reduces to QALYs if $Y$ fails to extend life over $\overline{X}$.

One can represent incremental HYT as

3 $$\Delta{\text{HYT}}\left( {Y,\overline{X}} \right) = \left\{ {\begin{array}{*{20}c} {\mathop \sum \limits_{t} \left( {S_{Yt} - S_{{\overline{X}t}} } \right) + \mathop \sum \limits_{t} \left( {S_{Yt} } \right)\left( {Q_{Yt} - Q_{{\overline{X}t}} } \right),} & {{\text{ if }}\mathop \sum \limits_{t} S_{{\overline{X}t}} < \mathop \sum \limits_{t} S_{Yt} } \\ {\mathop \sum \limits_{t} \left\{ {S_{Yt} Q_{Yt} - S_{{\overline{X}t}} Q_{{\overline{X}t}} } \right\},} & {{\text{ if }}\mathop \sum \limits_{t} S_{{\overline{X}t}} \ge \mathop \sum \limits_{t} S_{Yt} } \\ \end{array} } \right..$$

We will focus primarily on the case of one standard-of-care comparator, where $\overline{X} = X^{*} = X$. In this case, incremental HYT adds a constant adjustment to incremental EVL, equal to the covariance between incremental survival and quality-of-life ($\sum\nolimits_{t} {\left( {S_{Yt} - S_{Xt} } \right)\left( {Q_{Yt} - Q_{Xt} } \right)}$).

An alternative approach to addressing discrimination is offered by generalized risk-adjusted cost-effectiveness (GRACE) [1, 17]. Bleichrodt and Quiggin recognized that the traditional CEA embeds a potentially nonlinear relationship between health and health-related utility [22]. GRACE explicitly models this nonlinear utility function, revealing how risky health outcomes, disease severity, and pre-existing disability influence value. Define $Q_{0}$ as QoL in the baseline pre-illness period. Traditional CEA often assumes that $Q_{0} = 1$, or that individuals find themselves in perfect health prior to illness [23]. In contrast, GRACE allows for the possibility that $Q_{0} < 1$, if, for example, the population of interest suffers from permanent disability or other pre-existing health limitations prior to the onset of the relevant illness. The GRACE metric can be expressed as [19, Eq. (22c)]

4 $$\Delta {\text{GRACE}}\left( {Y,X;Q_{0} } \right) = \mathop \sum \limits_{t} \left[ {\left( {S_{Yt} - S_{Xt} } \right)\frac{{W\left( {Q_{Yt} } \right)}}{{W\left( {Q_{0} } \right)}} + S_{Xt} \frac{{W\left( {Q_{Yt} } \right) - W\left( {Q_{Xt} } \right)}}{{W\left( {Q_{0} } \right)}}} \right].$$

Here, $W$ represents the consumer’s utility over health-related quality-of-life. To facilitate comparison, $\Delta {\text{GRACE}}$ is expressed here in units that have the same incremental monetary value as a QALY, even though GRACE does not require the use of QALYs as a metric of benefit. Under GRACE, pre-existing disability affects both the marginal utility of consumption, $U^{\prime}\left( c \right)W\left( {Q_{0} } \right)$, and the value of life-extension among disabled persons. When $W$ exhibits constant relative risk-aversion (CRRA), GRACE ensures permanent disability has no effect on the value of life-extension in the next period [1]. Roughly speaking, under CRRA, degradations in utility due to permanent disability exactly offset increase in the willingness to pay for this family of models, thereby treating all persons equitably.

### Generic principles for consistent healthcare resource allocation

There are many different healthcare allocation methods, each with its own mathematical formula. Rather than a single best approach, we present several generic principles for healthcare resource allocation. The principles are normative in the sense that each provides a rationale to justify or to criticize approaches to value assessment. They are generic in that their properties are desirable in any objective function that aims to allocate healthcare resources. Although this set of generic principles is too narrow to justify any one formula, it is broad enough to rule out some formulae on grounds that they violate normative principles.

Define $\Delta {\text{Metric}}\left( {Y,X} \right)$ as some metric computing the incremental benefit of $Y$ over $X$; this could be $\Delta {\text{QALY}}$, $\Delta {\text{EVL}}$, $\Delta {\text{HYT}}$, $\Delta {\text{GRACE}}$, or another such metric. The principles below set forth axiomatic requirements for metrics that support logically consistent, non-discriminatory value assessment. We offer these as arguably “necessary” conditions, without claiming that they are both necessary and sufficient to define a single measurement approach.

#### The principle of additive value

We begin with a choice axiom that dates at least to work conducted in the 1950s by the philosopher, Donald Davidson, and the economist, Jacob Marschak. They demonstrated that well-defined utility functions over possibly stochastic choice alternatives required the “Quadruple Condition” [24, p. 420, 25, p. 132]. Formally, define health interventions, $A, B, C, D$. A metric satisfies the “Quadruple Condition” when

$$\Delta {\text{Metric}}\left( {A,B} \right) \ge \Delta {\text{Metric}}\left( {C,D} \right){\text{ implies }} \Delta {\text{Metric}}\left( {A,C} \right) \ge \Delta {\text{Metric}}\left( {B,D} \right).\quad \quad ({\text{Principle}}\quad 1)$$

An example illustrates its importance for well-defined utility functions. Suppose $\Delta {\text{Metric}}$ permits the well-defined calculation of marginal utility and thus monetary value, for health interventions, $A$, $B$, $C$, and $D$. Therefore, define $V_{A}$, $V_{B}$, $V_{C}$, and $V_{D}$ as the value of interventions $A$, $B$, $C$, and $D$, respectively. Suppose without loss of generality that $V_{A} - V_{B} > V_{C} - V_{D}$. It must then be true that $V_{A} - V_{C} > V_{B} - V_{D}$. This logical relationship follows from the quadruple condition. Moreover, since this condition requires additivity of value, we henceforth refer to it as “The Principle of Additive Value.”

**Fig. 1:**
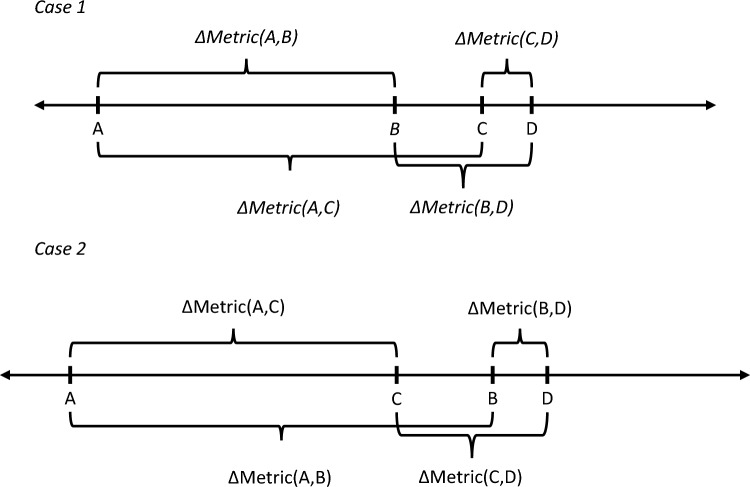
Representing Principle (1), the principle of additive value

Since distances on a straight line always satisfy additive incremental value, Fig. 1 is a useful example of its meaning. Note the figure depicts two cases. The first is $A > B > C > D$ and the second is $A > C > B > D$. In Case 1, additive incremental value ensures that the implied $\Delta {\text{Metric}}\left( {B,C} \right)$ remains the same when defined as $\Delta {\text{Metric}}\left( {B,C} \right) \equiv \Delta {\text{Metric}}\left( {A,C} \right) - \Delta {\text{Metric}}\left( {A,B} \right)$, or as $\Delta {\text{Metric}}\left( {B,C} \right) = \Delta {\text{Metric}}\left( {B,D} \right) - \Delta {\text{Metric}}\left( {C,D} \right)$. Similarly, in Case 2, the implied $\Delta {\text{Metric}}\left( {C,B} \right)$ remains the same when defined as $\Delta {\text{Metric}}\left( {C,B} \right) = \Delta {\text{Metric}}\left( {A,B} \right) - \Delta {\text{Metric}}\left( {A,C} \right)$ or as $\Delta {\text{Metric}}\left( {C,B} \right) = \Delta {\text{Metric}}\left( {B,D} \right) - \Delta {\text{Metric}}\left( {C,D} \right)$. This property allows the analyst to add up incremental value calculations involving overlapping technologies, and it also permits calculation of incremental value for two technologies that were compared to two different standards of care.

Practitioners of cost-effectiveness may be familiar with a special case of Principle (1), the approach of “Indirect Comparison.” For arbitrary interventions, $X$, $Y$, and $Z$, this requires

$$\Delta {\text{Metric}}\left( {Z,X} \right) \ge \Delta {\text{Metric}}\left( {Y,X} \right)\,\, {\text{if and only if }} \Delta {\text{Metric}}\left( {Z,Y} \right) \ge 0.\quad \quad ({\text{Condition}}\;1)$$

Condition (1), of “Indirect Comparison,” is the special case of Principle (1) that occurs when health interventions $B$ and $D$ are identical. If the incremental value of $Z$ exceeds the incremental value of $Y$, with respect to a uniform comparator, then $Z$ must produce incremental value over $Y$. If this condition fails, the decision maker cannot rely on indirect comparisons to rank-order technologies and must instead conduct direct pairwise comparisons of all technology pairs. Since the rank-ordering of health interventions is an essential function of economic evaluation [26], indirect comparison is an essential feature of value metrics.

#### The principle that life-extension does no harm

Next, we require that higher standard-of-care survival is at least as preferable as lower standard-of-care survival. (Here and elsewhere, we present our principles in terms of standard-of-care survival gains, rather than novel technology survival gains, but they are meant to apply to both.). The economics literature implies weakly positive value of survival, for health states weakly better than death, under plausible conditions nearly always required by applied economists [27]^3^: (1) period utility strictly increases in consumption, and (2) period utility is nonnegative. To formalize this principle, consider a prior and a current standard-of-care intervention, $L$ and $L^{\prime}$, that produce identical quality of life, $\left\{ {Q_{t} } \right\}_{t = 1}^{T}$. In addition, assume that $S_{{L^{\prime}t}} \ge S_{Lt}$ for all *t*, and that there is at least one period, $t^{\prime}$, such that $S_{{L^{\prime}t^{\prime}}} > S_{{Lt^{\prime}}}$. For any novel intervention, $Z$, this principle then requires that

$$\Delta {\text{Metric}}\left( {Z,L^{\prime}} \right) \le \Delta {\text{Metric}}\left( {Z,L} \right).\quad \quad ({\text{Principle}}\;2)$$

Strictly higher standard-of-care survival, holding quality-of-life constant, weakly reduces the incremental value of any fixed novel technology.

#### The principle of bounded average value from life-extension

Heuristically, “small” changes in total survival should not produce “large” changes in value, a premise proposed in earlier work by Moreno-Ternero and Østerdal [14]. We operationalize this principle by requiring that average value or slope, $\frac{{\Delta {\text{Value}}}}{{\Delta {\text{Survival}}}}$, is bounded uniformly above, when quality-of-life profiles are held constant. Failure of this principle permits the conclusion that unbounded amounts of consumption should be given up in exchange for negligibly small amounts of survival. For any quality-of-life profiles $\left\{ {Q_{Lt} } \right\}$ and $\left\{ {Q_{Zt} } \right\}$, we require the existence of some positive real number, $\overline{V} \in {\mathbb{R}}$, such that for any pair of technologies $L^{\prime}$ and $L$ with quality-of-life profile $\left\{ {Q_{Lt} } \right\}$ and survival profiles satisfying $\mathop \sum \limits_{t} S_{{L^{\prime}t}} > \mathop \sum \limits_{t} S_{Lt}$, and any third (distinct) novel technology $Z$ with quality-of-life profile $\left\{ {Q_{Zt} } \right\}$ and an arbitrary survival profile

$$\left| {\frac{{\left[ {\Delta {\text{Metric}}\left( {Z,L} \right) - \Delta {\text{Metric}}\left( {Z,L^{\prime}} \right)} \right]}}{{\mathop \sum \nolimits_{t} S_{{L^{\prime}t}} - \mathop \sum \nolimits_{t} S_{Lt} }}} \right| < \overline{V.} \quad \quad ({\text{Principle}}\;{3}).$$

In words, holding quality-of-life profiles constant, the average value of standard-of-care survival gains must be uniformly bounded. This ensures that “small” standard-of-care survival gains do not lead to “large” changes in value.^4^

#### The principle of convex survival preferences

Even though standard-of-care therapy evolves, value metrics ought to produce consistent implications over time. If a novel therapy, $Z$, is judged superior to both a previous standard-of-care, $X$, and a current standard-of-care, $Y$, then it should also be judged superior to mixed strategies that use these former and current standard-of-care therapies in conjunction. Suppose there are technologies, $X$, $Y$, and $Z$, and some $0 < p < 1$, where $X$ and $Y$ are current and former standard-of-care therapies producing equal quality-of-life. Define $C$ as the intervention that represents a “convex combination” of $X$ and $Y$, in the sense that $C$ generates survival probabilities and quality-of-life weights, $\left\{ {pS_{Xt} + \left( {1 - p} \right)S_{Yt} ;pQ_{Xt} + \left( {1 - p} \right)Q_{Yt} } \right\}_{t = 1}^{T}$, for some $0 < p < 1$. Convex survival preferences require

$${\text{If }} \Delta {\text{Metric}}\left( {Z,X} \right) > 0 \;{\text{and}}\; \Delta {\text{Metric}}\left( {Z,Y} \right) > 0\;{\text{then}}\;\Delta {\text{Metric}}\left( {Z,C} \right) > 0.\quad \quad ({\text{Principle}}\;{4})$$

The principle will also hold in reverse: two superior standard-of-care technologies do not become inferior simply when combined in a convex fashion. Significantly, this principle must also be satisfied for the incremental metric to satisfy the conditions of neoclassical expected utility theory, which imply that any lottery over $Y$ and $Z$ be preferable to receiving $X$ for certain.

#### The principle of non-discrimination against the sick and disabled

Considering the purpose of our analysis, we seek metrics that avoid “health- and disability-discrimination.” The US Inflation Reduction Act stipulates that CMS cannot evaluate medicines “in a manner that treats extending the life of an elderly, disabled, or terminally ill individual as of lower value than extending the life of an individual who is younger, non-disabled, or not terminally ill.”^5^ We require that an increase in survival ought to be at least as valuable for older, more disabled, or terminally ill groups as for their younger, less disabled, or not terminally ill counterparts. Recall the definition of $Q_{0}$, the pre-existing quality-of-life level, prior to the onset of illness. $Q_{0}$ will reflect age, disability, and any other pre-existing comorbidities prior to the onset of the relevant illness. Consider two groups of patients, group $L$ (for “low” quality-of-life) and group $H$ (for “high” quality-of-life), with initial quality-of-life levels $Q_{0}^{L}$ and $Q_{0}^{H}$, where $0 < Q_{0}^{L} < Q_{0}^{H} \le 1$. We wish to consider a health intervention that increases survival in both groups by the same amount, while holding quality-of-life constant. Formally, suppose $I_{L}$ and $I_{H}$ are standard-of-care interventions used on groups $L$ and $H$, respectively. They produce quality-of-life sequences $\left\{ {Q_{t}^{L} } \right\}_{t = 1}^{T}$ and $\left\{ {Q_{t}^{H} } \right\}_{t = 1}^{T}$, where $Q_{t}^{L} < Q_{t}^{H}$ $\forall t$, and survival sequences $\left\{ {S_{t}^{L} } \right\}_{t = 1}^{T}$ and $\left\{ {S_{t}^{H} } \right\}_{t = 1}^{T}$, where $S_{t}^{L} \le S_{t}^{H}$ $\forall t$. Furthermore, there are novel interventions, $I_{L}{\prime}$ and $I_{H}{\prime}$ also used on groups $L$ and $H$. They produce the same quality-of-life sequences as the standard of care. Their survival sequences, $\left\{ {S_{t}^{{L^{\prime}}} } \right\}_{t = 1}^{T}$ and $\left\{ {S_{t}^{{H^{\prime}}} } \right\}_{t = 1}^{T}$ are different in the following sense: $\exists t^{\prime}$, such that $S_{{t^{\prime}}}^{{L^{\prime}}} - S_{{t^{\prime}}}^{L} = S_{{t^{\prime}}}^{{H^{\prime}}} - S_{{t^{\prime}}}^{H} = {\Delta } > 0$; $\forall t \ne t^{\prime}$, $S_{t}^{{L^{\prime}}} = S_{t}^{L}$ and $S_{t}^{{H^{\prime}}} = S_{t}^{H}$. We can then state our non-discrimination principle as

$$\Delta {\text{Metric}}\left( {H^{\prime},H} \right) = \Delta {\text{Metric}}\left( {L^{\prime},L} \right).\quad \quad 
({\text{Principle}}\;{5})$$

Intuitively, a given increase in survival should be equally valuable to the group with higher baseline quality-of-life as the group with lower baseline quality-of-life.

Finally, we note a useful implication if Principles (1) and (5) both hold. Together, these imply that increases in standard-of-care survival must be valuable either for both $H$ and $L$ patients or for neither type of patient. Specifically, for any novel intervention $Y$, it must be true that^6^

$$\Delta {\text{Metric}}\left( {Y,H} \right) \ge \Delta {\text{Metric}}\left( {Y,H^{\prime}} \right) \Rightarrow \Delta {\text{Metric}}\left( {Y,L} \right) \ge \Delta {\text{Metric}}\left( {Y,L^{\prime}} \right).\quad \quad ({\text{Condition}}\;{2})$$

The proof of Condition (2) begins with an implication of Principle (1)^7^

5 $$\Delta {\text{Metric}}\left( {Y,H} \right) \ge \Delta {\text{Metric}}\left( {Y,H^{\prime}} \right) \Rightarrow \Delta {\text{Metric}}\left( {Y,Y} \right) \ge \Delta {\text{Metric}}\left( {H,H^{\prime}} \right).$$

By Principle (5), $\Delta {\text{Metric}}\left( {H,H^{\prime}} \right) = \Delta {\text{Metric}}\left( {L,L^{\prime}} \right)$,^8^ so $\Delta {\text{Metric}}\left( {Y,Y} \right) \ge \Delta {\text{Metric}}\left( {L,L^{\prime}} \right)$. Furthermore, applying Principle (1) again implies

6 $$\Delta {\text{Metric}}\left( {Y,Y} \right) \ge \Delta {\text{Metric}}\left( {L,L^{\prime}} \right) \Rightarrow \Delta {\text{Metric}}\left( {Y,L} \right) \ge \Delta {\text{Metric}}\left( {Y,L^{\prime}} \right).$$

The resulting chain of inequalities then proves Condition (2) as a consequence of Principles (1) and (5).

## Principle violations within the EVL and HYT metrics

We now show the existence of health intervention decisions, such that incremental EVL and HYT: (1) violate both the principle of additive value (Principle 1) and the condition enabling indirect comparison (Condition 1); (2) violate either (a) the principle that life-extension does no harm (Principle 2) and the condition of non-discrimination for standard-of-care survival gains (Condition 2), or (b) the principle of bounded value from life-extension (Principle 3); and (3) violate the principle of convex survival preferences (Principle 4).

### Failures of additivity in EVL and HYT

Incremental EVL and HYT fail to obey additivity when the metrics are employed over time and across different eras in standard-of-care technology. This results in violation of additive value (Principle 1) and indirect comparison (Condition 1).

#### Numerical examples

Numerical examples illustrate the intuition. Consider the standard-of-care therapy, $X$, with the three-period survival and quality-of-life sequences $\left\{ {S_{Xt} } \right\} = \left\{ {0.1, 0.1, 0.1} \right\}$ and $\left\{ {Q_{Xt} } \right\} = \left\{ {0.2, 0.2, 0.1} \right\}$. $X$ offers limited survival and quality-of-life prospects. A new therapy, $Y$, launches, with much improved survival and quality-of-life sequences $\left\{ {S_{Yt} } \right\} = \left\{ {0.9, 0.9, 0.9} \right\}$, and $\left\{ {Q_{Yt} } \right\} = \left\{ {0.7, 0.9, 1.0} \right\}$. EVL implies that the new therapy provides an advance over the current standard-of-care, with $\Delta {\text{EVL}}\left( {Y,X} \right) = 2.61$. Next year, another technology emerges in the research pipeline, $Z$, with survival and quality-of-life sequences $\left\{ {S_{Zt} } \right\} = \left\{ {0.99, 0.99, 0.99} \right\}$, and $\left\{ {Q_{Zt} } \right\} = \left\{ {0.7, 0.5, 1.0} \right\}$. If eventually approved, $Z$ would offer longer total survival than $Y$, and EVL indeed concludes that it will be an even bigger advance over the current standard-of-care, where $\Delta {\text{EVL}}\left( {Z,X} \right) = 2.84$. By the time $Z$ launches, however, $Y$ has become the sole standard-of-care, which reverses the earlier conclusion about the value of $Z$ over $Y$. Even though EVL implied that $Z$ represented a bigger advance over $X$ than $Y$ did, once the standard-of-care switches to $Y$, EVL selects $Y$ over $Z$, because $\Delta {\text{EVL}}\left( {Z,Y} \right) = - 0.09$, leading to an inconsistent conclusion.

This occurs, because the shift in the standard-of-care changes the “reference” survival profile that EVL uses to calculate the expected value of quality-of-life improvements. Technology $Y$’s quality-of-life advantage over technology $Z$ becomes more prominent when the standard-of-care survival rises from the low level afforded by $X$ to the higher level afforded by $Y$.

HYT suffers from an analogous problem. Continue with the same example technologies from above. Initially, $X$ is the sole standard-of-care technology and $Y$ is a novel technology, so $\Delta {\text{HYT}}\left( {Y,X} \right) = 4.29$. Later, $Y$ joins $X$ as another standard-of-care option, and $Z$ emerges as a novel technology. In this later time period, $\Delta {\text{HYT}}\left( {Z,X} \right) = 4.353$, but head-to-head comparison of $Z$ and $Y$ reveals that $\Delta {\text{HYT}}\left( {Z,Y} \right) = - 0.126.$ Using the original comparison of $Y$ to $X$ implies that $Y$ is a smaller advance than $Z$, in the sense that $\Delta {\text{HYT}}\left( {Z,X} \right) > \Delta {\text{HYT}}\left( {Y,X} \right)$. However, subsequent head-to-head comparison concludes that $Y$ dominates $Z$. With HYT, technology $Y$’s quality-of-life advantage over technology $Y$ becomes accentuated when the reference survival rises from the level provided by $Y$ to the slightly higher level provided by $Z$.

In both these cases, the shift in the reference therapy causes EVL and HYT to violate the critical principle of additivity for comparisons made over time. Using EVL as an example, since $\Delta {\text{EVL}}\left( {Y,X} \right) = 2.61$ and $\Delta {\text{EVL}}\left( {Z,X} \right) = 2.84$, additivity would imply that $\Delta {\text{EVL}}\left( {Z,Y} \right) = \Delta {\text{EVL}}\left( {Z,X} \right) - \Delta {\text{EVL}}\left( {Y,X} \right) = 0.23$, but $\Delta {\text{EVL}}\left( {Z,Y} \right)$ arrives not only at a different number but even at the opposite conclusion for technology adoption.

#### Proving the failure of additivity

The numerical example motivates the following theorem.

##### Theorem 1

(Failure of additive incremental value) *There exist health interventions* $A$, $B$, $C$, and $D$, *such that* $\Delta {\text{EVL}}\left( {A,B} \right) > \Delta {\text{EVL}}\left( {C,D} \right)$, *but* $\Delta {\text{EVL}}\left( {A,C} \right) < \Delta {\text{EVL}}\left( {B,D} \right)$. *There exist interventions* $A$, $B$, $C$, *and* $D$, *such that* $\Delta {\text{HYT}}\left( {A,B} \right) > \Delta {\text{HYT}}\left( {C,D} \right)$ *but* $\Delta {\text{HYT}}\left( {A,C} \right) < \Delta {\text{HYT}}\left( {B,D} \right)$. *Therefore*, $\Delta {\text{EVL}}$ *and* $\Delta {\text{HYT}}$ *violate Principle* (1).

The proof, which appears in the appendix, relies on changes over time in the standard-of-care therapy, similar to the intuition illustrated in the numerical example above. An immediate corollary is the failure of both EVL and HYT to satisfy the Condition enabling Indirect Comparison. The following statement, proven in the appendix, formalizes this point, which follows as a simple consequence of the case where technologies $B$ and $D$ are the same.

##### Corollary 1.1

(Failure of indirect comparison) *There exist health interventions* $A$, $B$, *and* $C$, *such that* $\Delta {\text{EVL}}\left( {A,C} \right) > \Delta {\text{EVL}}\left( {B,C} \right)$, *but* $\Delta {\text{EVL}}\left( {A,B} \right) < 0$. *There exist interventions* $A$, $B$, *and* $C$, *such that* $\Delta {\text{HYT}}\left( {A,C} \right) > \Delta {\text{HYT}}\left( {B,C} \right)$, *but* $\Delta {\text{HYT}}\left( {A,B} \right) < 0$. *Therefore*, $\Delta {\text{EVL}}$ *and* $\Delta {\text{HYT}}$ *violate Condition* (1).

Therefore, neither EVL nor HYT can be used reliably over time to conduct indirect comparisons or compute value-based prices. In practice, all prior EVL and HYT comparisons would need to be updated every time there is an evolution in the standard-of-care, and the metrics maintain additivity only within a fixed standard-of-care era.

### Failures due to discontinuity in EVL and HYT

The piecewise structure of $\Delta {\text{EVL}}$ and $\Delta {\text{HYT}}$ causes them to switch calculation methods in the neighborhood where total survival is equal for the novel intervention and its standard of care. This switch creates the possibility of discontinuity in total survival in this neighborhood, a discontinuity that results in three additional violations.

#### Numerical examples of violations due to discontinuity

A simple two-period example illustrates the intuition. Suppose novel intervention $Y$ exhibits the two-period sequence of survival probabilities, $\left\{ {S_{Y1} = 0.8, S_{Y2} = 0.7} \right\}$. Meanwhile, the standard-of-care technology evolves. Denote the original standard-of-care as $X_{A}$ and the new one as $X_{B}$. Technology $X_{A}$ produces survival $\left\{ {S_{XA1} = 0.9, S_{XA2} = 0.595} \right\}$, while $X_{B}$ produces $\left\{ {S_{XB1} = 0.9, S_{XB2} = 0.605} \right\}$. The novel intervention produces quality-of-life, $\left\{ {Q_{Y1} = 0.05,Q_{Y2} = 0.16} \right\}$, while the two standard-of-care therapies produce the same quality-of-life $\left\{ {Q_{X1} = 0.1,Q_{X2} = 0.1} \right\}$. Notice that $X_{B}$ strictly dominates $X_{A}$, because it produces strictly more total survival, weakly more survival in each period, and identical quality-of-life in each period. However, the EVL metric implies $\Delta {\text{EVL}}\left( {Y,X_{A} } \right) = - 0.0043$ and $\Delta {\text{EVL}}\left( {Y,X_{B} } \right) = 0.0015$, calculated when $X_{A}$ is the standard-of-care and when $X_{B}$ is the standard of care, respectively. According to the EVL measurement strategy, $Y$ is worse than $X_{A}$ but better than its successor, $X_{B}$, even though $X_{B}$ strictly dominates $X_{A}$.^9^ Because EVL switches between QALYs and its own ad hoc metric, the result is a flawed decision outcome violating the principle that life-extension does no harm (Principle 2).

Ordinarily, the incremental value of a novel technology will decrease continuously and monotonically in total standard-of-care survival, all else equal. However, the piecewise structure of the incremental EVL metric creates a possible discontinuity at the point where the EVL formula switches its calculation methodology. The same pathology arises for HYT, because it too switches between QALY measurement and its own ad hoc metric. Keep the survival probabilities the same as in the example above, but now alter the quality-of-life profiles to be: $\left\{ {Q_{X1} = 0.06,Q_{X2} = 0.17} \right\}$ and $\left\{ {Q_{Y1} = 0.105,Q_{Y2} = 0.105} \right\}$. The HYT metric implies $\Delta {\text{HYT}}\left( {Y,X_{A} } \right) = - 0.0045$ and $\Delta {\text{HYT}}\left( {Y,X_{B} } \right) = 0.00065$, calculated in the $X_{A}$ era and the $X_{B}$ era, respectively. Again, $Y$ is worse than $X_{A}$ but better than $X_{B}$, even though $X_{B}$ strictly dominates $X_{A}$.

The numerical examples also illustrate how a type of disability-discrimination can arise from negative survival values. Suppose that the standard-of-care technologies above are given to patients with higher quality-of-life sequences equal to $\left\{ {Q_{X1} = 0.7,Q_{X2} = 0.7} \right\}$. Now, $\Delta {\text{EVL}}\left( {Y,X_{A} } \right) = - 0.9013 > - 0.9015 = \Delta {\text{EVL}}\left( {Y,X_{B} } \right)$. Therefore, the increase in standard-of-care survival produces positive value to patients at quality-of-life level $0.7$, but not to patients at quality-of-life level $0.1$. Similarly, at this higher quality-of-life level for standard-of-care patients, $\Delta {\text{HYT}}\left( {Y,X_{A} } \right) = - 0.8875 > - 0.896 = \Delta {\text{HYT}}\left( {Y,X_{B} } \right)$.

#### Proving principle violations in EVL and HYT that result from discontinuity

Consider a novel intervention, $Y$, along with a time-series of standard-of-care therapies, $X\left( \delta \right)$, where $\delta$ is an index of technological progress in the standard-of-care. The novel intervention, $Y$, is characterized by $\left\{ {S_{Yt} ;Q_{Yt} } \right\}_{t = 1}^{T}$. The standard-of-care therapies, $X\left( \delta \right)$, differ only in survival, with health outcomes characterized by $\left\{ {S_{Xt} \delta ;Q_{Yt} } \right\}_{t = 1}^{T}$, where $0 \le \delta \le 1$ represents the increase in survival due to technological change over time. Moreover, suppose $X$ has strictly higher total survival than $Y$.

Define the point $\delta^{*} \equiv \frac{{\mathop \sum \nolimits_{t} S_{Yt} }}{{\mathop \sum \nolimits_{t} S_{Xt} }} < 1.$ Notice that $\mathop \sum \limits_{t} S_{Xt} > \mathop \sum \limits_{t} S_{Yt}$, but $\mathop \sum \limits_{t} S_{Yt} \ge \mathop \sum \limits_{t} \left( {S_{Xt} {\updelta }} \right) \forall \delta \le \delta^{*}$. Since $\sum\nolimits_{t} {S_{Xt} \delta^{*} } = \sum\nolimits_{t} {S_{Yt} }$, $\delta^{*}$ is the technology level at which the incremental EVL metric switches its formula. Since the calculation method discretely switches at $\delta^{*}$, $\Delta {\text{EVL}}$ can often be discontinuous in total standard-of-care survival at $\delta^{*}$. To begin our proof of this point, note the following:

7 $$L_{{{\text{EVL}}}}^{*} \equiv \mathop {\lim }\limits_{{\delta \to \delta^{* - } }} \Delta {\text{EVL}}\left( {Y,X\left( \delta \right)} \right) = \mathop \sum \limits_{t} S_{Xt} \delta^{*} \left( {Q_{Yt} - Q_{Xt} } \right)$$

8 $$R_{{{\text{EVL}}}}^{*} \equiv \mathop {\lim }\limits_{{\delta \to \delta^{* + } }} \Delta {\text{EVL}}\left( {Y,X\left( \delta \right)} \right) = \mathop \sum \limits_{t} S_{Yt} Q_{Yt} - S_{Xt} \delta^{*} Q_{Xt}$$

9 $$R_{{{\text{EVL}}}}^{*} - L_{{{\text{EVL}}}}^{*} = \mathop \sum \limits_{t} \left( {S_{Yt} - S_{Xt} \delta^{*} } \right)Q_{Yt} .$$

Mechanically, discontinuity exists at $\delta^{*}$ if the right-hand and left-hand limits, $R_{{{\text{EVL}}}}^{*}$ and $L_{{{\text{EVL}}}}^{*}$, are strictly unequal at $\delta^{*}$. If $Q_{Yt}$ is constant over time, it is obvious from Eq. (9) that there is no such discontinuity, because $\sum\nolimits_{t} {\left( {S_{Yt} - S_{Xt} \delta^{*} } \right) = 0}$, and thus, $R_{{{\text{EVL}}}}^{*} = L_{{{\text{EVL}}}}^{*}$. However, this is a special case. In general, the difference, $R_{{{\text{EVL}}}}^{*} - L_{{{\text{EVL}}}}^{*}$, equals $\mathop \sum \limits_{t} \left( {S_{Yt} - S_{Xt} \delta^{*} } \right)Q_{Yt}$, the covariance between $Q_{Yt}$ and $S_{Yt} - S_{Xt} \delta^{*}$. This covariance that can be positive, negative, or zero. $\Delta {\text{EVL}}$ is continuous at $\delta^{*}$ only if incremental survival and quality of life under the intervention are exactly orthogonal over time, i.e., if $\sum\nolimits_{t} {\left( {S_{Yt} - S_{Xt} \delta^{*} } \right)Q_{Yt} = 0}$. If orthogonality fails, so will the continuity of $\Delta {\text{EVL}}$ in total standard-of-care survival.

Since HYT also switches measurement methods at the point where $\sum\nolimits_{t} {S_{Yt} } = \sum\nolimits_{t} {S_{Xt} \delta^{*} }$, it inherits the same discontinuity, albeit with a few minor technical differences. The jump in the incremental HYT function at the discontinuity can be characterized as

10 $$L_{{{\text{HYT}}}}^{*} \equiv \mathop {\lim }\limits_{{\delta \to \delta^{* - } }} \Delta {\text{HYT}}\left( {Y,X\left( \delta \right)} \right) = \mathop \sum \limits_{t} S_{Yt} \left( {Q_{Yt} - Q_{Xt} } \right)$$

11 $$R_{{{\text{HYT}}}}^{*} \equiv \mathop {\lim }\limits_{{\delta \to \delta^{* + } }} \Delta {\text{HYT}}\left( {Y,X\left( \delta \right)} \right) = \mathop \sum \limits_{t} S_{Yt} Q_{Yt} - S_{Xt} \delta^{*} Q_{Xt}$$

12 $$R_{{{\text{HYT}}}}^{*} - L_{{{\text{HYT}}}}^{*} = \mathop \sum \limits_{t} \left( {S_{Yt} - S_{Xt} \delta^{*} } \right)Q_{Xt} .$$

Comparing Eqs. (9) and (12) reveals a small modification. The existence and direction of the discontinuity at $\delta^{*}$ depends on the covariance between $Q_{Xt}$ (rather than $Q_{Yt}$) and $S_{Yt} - S_{Xt} \delta^{*}$, a covariance that can again be positive, negative, or zero.

**Fig. 2:**
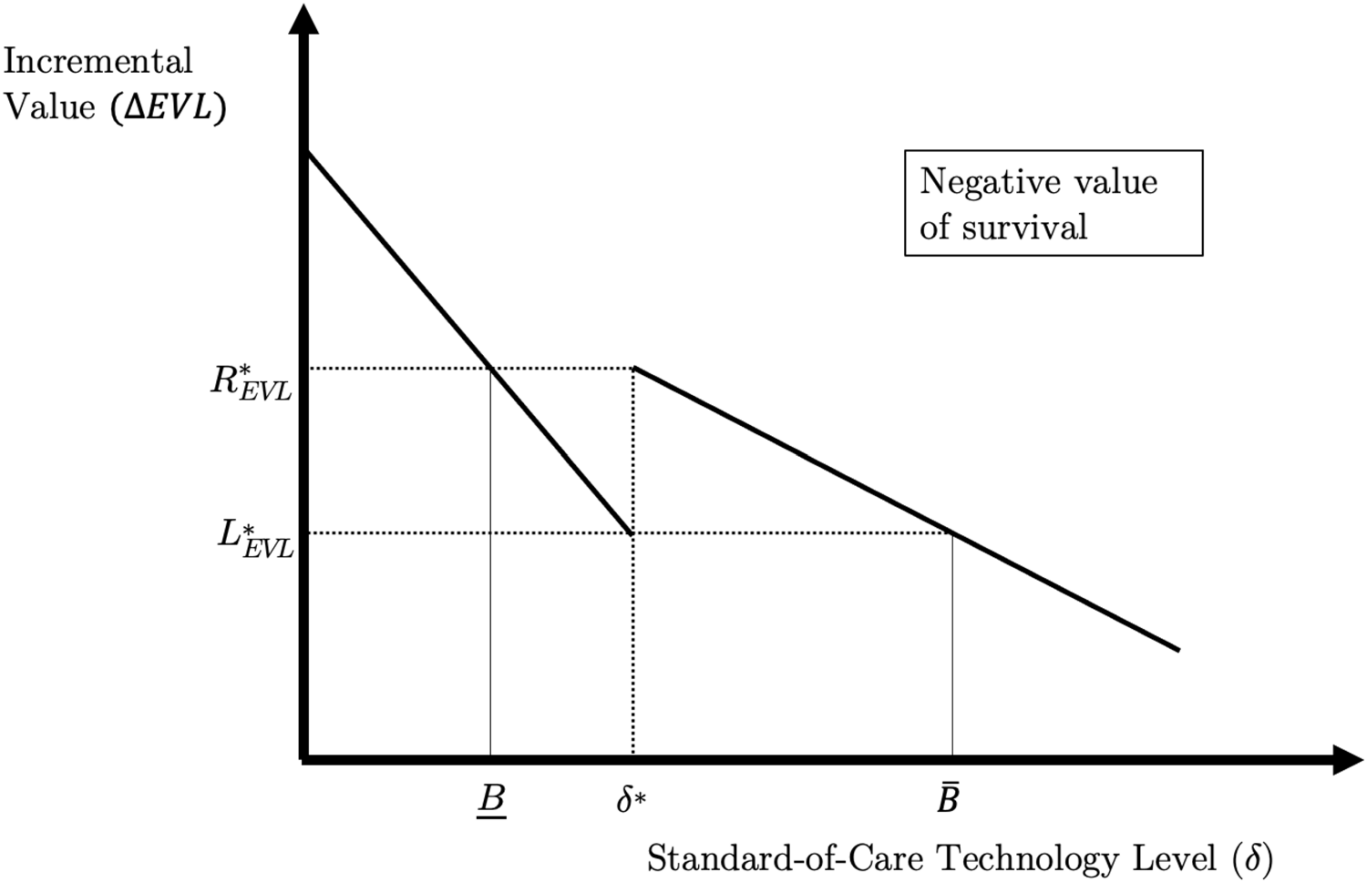
The case of negatively valued survival gains under EVL and HYT. Notes: The figure illustrates the relationship between $\delta$ and $\Delta {\text{EVL}}\left( {Y,X\left( \delta \right)} \right)$ under the case where $R^{*} > L^{*}$. Incremental quality-of-life is held constant throughout the figure. An analogous figure obtains for $\Delta {\text{HYT}}\left( {Y,X\left( \delta \right)} \right)$, but where $R_{{{\text{HYT}}}}^{*} < L_{{{\text{HYT}}}}^{*}$

*Negative value of survival under EVL and HYT* When the right-hand limit exceeds the left-hand limit ($R_{{{\text{EVL}}}}^{*} > L_{{{\text{EVL}}}}^{*}$), there can be negative value of survival under EVL. Figure 2 illustrates this case graphically, where the *x*-axis measures $\delta$ and the *y*-axis measures incremental EVL.^10^ An analogous graphical relationship obtains for $\Delta {\text{HYT}}$ when $R_{{{\text{HYT}}}}^{*} > L_{{{\text{HYT}}}}^{*}$. Discontinuity in total survival means that the incremental value function will “jump” up or down. If it jumps up, as in this figure, an increase in standard-of-care survival will counter-intuitively increase the incremental value of the novel intervention.

Under $\Delta {\text{EVL}}$, negative values of survival gains are possible for values of $\delta$ within the interval, $\left[ {\underline {B}_{{{\text{EVL}}}} ,\overline{B}_{{{\text{EVL}}}} } \right]$. The endpoints of this interval can be defined implicitly as

13 $$\underline {B}_{{{\text{EVL}}}} : \mathop \sum \limits_{t} \left( {S_{Yt} - S_{Xt} \underline {B}_{{{\text{EVL}}}} } \right) + \mathop \sum \limits_{t} S_{Xt} \underline {B}_{{{\text{EVL}}}} \left( {Q_{Yt} - Q_{Xt} } \right) = R_{{{\text{EVL}}}}^{*}$$

14 $$\overline{B}_{{{\text{EVL}}}} : \mathop \sum \limits_{t} \left( {S_{Yt} Q_{Yt} - S_{Xt} \overline{B}_{{{\text{EVL}}}} Q_{Xt} } \right) = L_{{{\text{EVL}}}}^{*} .$$

Since $\Delta {\text{EVL}}$ is monotonic decreasing on $\left[ {0,\delta^{*} } \right)$ and $\left( {\delta^{*} ,1} \right]$, so long as $L_{{{\text{EVL}}}}^{*} < R_{{{\text{EVL}}}}^{*}$, it follows that $\underline {B}_{{{\text{EVL}}}} < \overline{B}_{{{\text{EVL}}}}$.

Analogously, incremental HYT produces negative values of survival over the interval, $\left( {\underline {B}_{{{\text{HYT}}}} ,\overline{B}_{{{\text{HYT}}}} } \right)$, where the endpoints are defined as follows:

15 $$\underline {B}_{{{\text{HYT}}}} : \mathop \sum \limits_{t} \left( {S_{Yt} - S_{Xt} \underline {B}_{{{\text{HYT}}}} } \right) + \mathop \sum \limits_{t} S_{Yt} \left( {Q_{Yt} - Q_{Xt} } \right) = R_{{{\text{HYT}}}}^{*}$$

16 $$\overline{B}_{{{\text{HYT}}}} : \mathop \sum \limits_{t} \left( {S_{Yt} Q_{Yt} - S_{Xt} \overline{B}_{{{\text{HYT}}}} Q_{Xt} } \right) = L_{{{\text{HYT}}}}^{*} .$$

This leads to the following theorem, proving that EVL and HYT violate Principle (2), the weakly positive value of survival gains.

##### Theorem 2

(Negative value of survival gains) *Suppose that* $T \ge 2$, $Y$ *is a novel intervention with survival and quality-of-life profiles* $\left\{ {S_{Yt} ;Q_{Yt} } \right\}_{t = 1}^{T}$, *and* $X\left( \delta \right)$ *is the family of standard-of-care interventions with profile* $\left\{ {S_{Xt} \delta ;Q_{Xt} } \right\}_{t = 1}^{T}$ where $\sum\nolimits_{t} {S_{Yt} } < \sum\nolimits_{t} {S_{Xt} }$

A) *Under these conditions, there exist standard-of-care interventions* $X\left( \phi \right)$ *and* $X\left( {\phi^{\prime}} \right)$, *such that* $X\left( {\phi^{\prime}} \right)$ *features strictly higher survival and weakly higher quality of life than* $X\left( \phi \right)$, *but that* $\Delta {\text{EVL}}\left( {Y,X\left( {\phi^{\prime}} \right)} \right) > \Delta {\text{EVL}}\left( {Y,X\left( \phi \right)} \right)$.
B) *There also exist standard-of-care interventions* $X\left( \rho \right)$ *and* $X\left( {\rho^{\prime}} \right)$, *such that* $X\left( {\rho^{\prime}} \right)$ *features strictly higher survival and weakly higher quality of life than* $X\left( \rho \right)$, *but that* $\Delta {\text{HYT}}\left( {Y,X\left( {\rho^{\prime}} \right)} \right) > \Delta {\text{HYT}}\left( {Y,X\left( \rho \right)} \right)$.

*Therefore, both* $\Delta {\text{EVL}}$ *and* $\Delta {\text{HYT}}$ *violate Principle* (2).

The proof appears in the appendix, but Fig. 2 illustrates the intuition. When $R^{*} > L^{*}$, there exists a region over which $\Delta {\text{EVL}}$ and $\Delta {\text{HYT}}$ can rise even though standard-of-care survival does too. Later, we consider the anomalies that arise if $R^{*} < L^{*}$.

*Disability-discrimination under EVL and HYT* Both EVL and HYT are engineered to satisfy Principle (5), equity for the disabled. However, their violation of monotonicity in survival leads them to violate Condition (2), which requires that standard-of-care survival gains should be valuable to the sick, whenever they are valuable to the healthy. This results in a type of disability-discrimination, which follows as a corollary of Theorem 2.

##### Corollary 2.1

(Disability discrimination) *Assume that* *Y* *and* $X\left( \delta \right)$ *are defined as described in the conditions of* Theorem 2, *and suppose* $\exists {{t^{\prime}}},$ *such that* $\frac{{S_{{Xt^{\prime}}} }}{{\mathop \sum \nolimits_{t} S_{Xt} }} > \frac{{S_{{Yt^{\prime}}} }}{{\mathop \sum \nolimits_{t} S_{Yt} }}$ *and* $Q_{{Xt^{\prime}}} \in \left( {0,1} \right)$. *Condition* (2) *is violated by both EVL and HYT, in the following way*.

A) *Suppose novel intervention* $Y$ *produces strictly higher total survival than standard-of-care* $X$, *but strictly lower survival than* $X$ *in at least one time period. Under these conditions, there exists another standard-of-care intervention*, $Z$, *producing the same survival profile as* $X$ *but strictly higher quality-of-life than* $X$, *and* $0 < \phi^{\prime\prime} < \phi^{\prime\prime\prime} < 1$, *where* $\Delta {\text{EVL}}\left( {Y,Z\left( {\phi^{\prime\prime}} \right)} \right) - \Delta {\text{EVL}}\left( {Y,Z\left( {\phi^{\prime\prime\prime}} \right)} \right) > 0$ *but* $\Delta {\text{EVL}}\left( {Y,X\left( {\phi^{\prime\prime}} \right)} \right) - \Delta {\text{EVL}}\left( {Y,X\left( {\phi^{\prime\prime\prime}} \right)} \right) < 0$.
B) *Alternatively, suppose novel intervention* $Y$ *produces strictly higher total survival than standard-of-care* $X$, *but strictly lower survival than* $X$ *in at least one time period. Under these conditions, there exists another standard-of-care intervention*, $Z$, *producing the same survival as* $X$ *but strictly higher quality-of-life than* $X$, *and* $0 < \phi^{\prime\prime} < \phi^{\prime\prime\prime} < 1$, *where* $\Delta {\text{HYT}}\left( {Y,Z\left( {\phi^{\prime\prime}} \right)} \right) - \Delta {\text{HYT}}\left( {Y,Z\left( {\phi^{\prime\prime\prime}} \right)} \right) > 0$ *but* $\Delta {\text{HYT}}\left( {Y,X\left( {\phi^{\prime\prime}} \right)} \right) - \Delta {\text{HYT}}\left( {Y,X\left( {\phi^{\prime\prime\prime}} \right)} \right) < 0.$

Under the relatively weak conditions of the corollary, Condition (2) is violated, and EVL results in a type of disability-discrimination. The same is true for HYT.

**Fig. 3:**
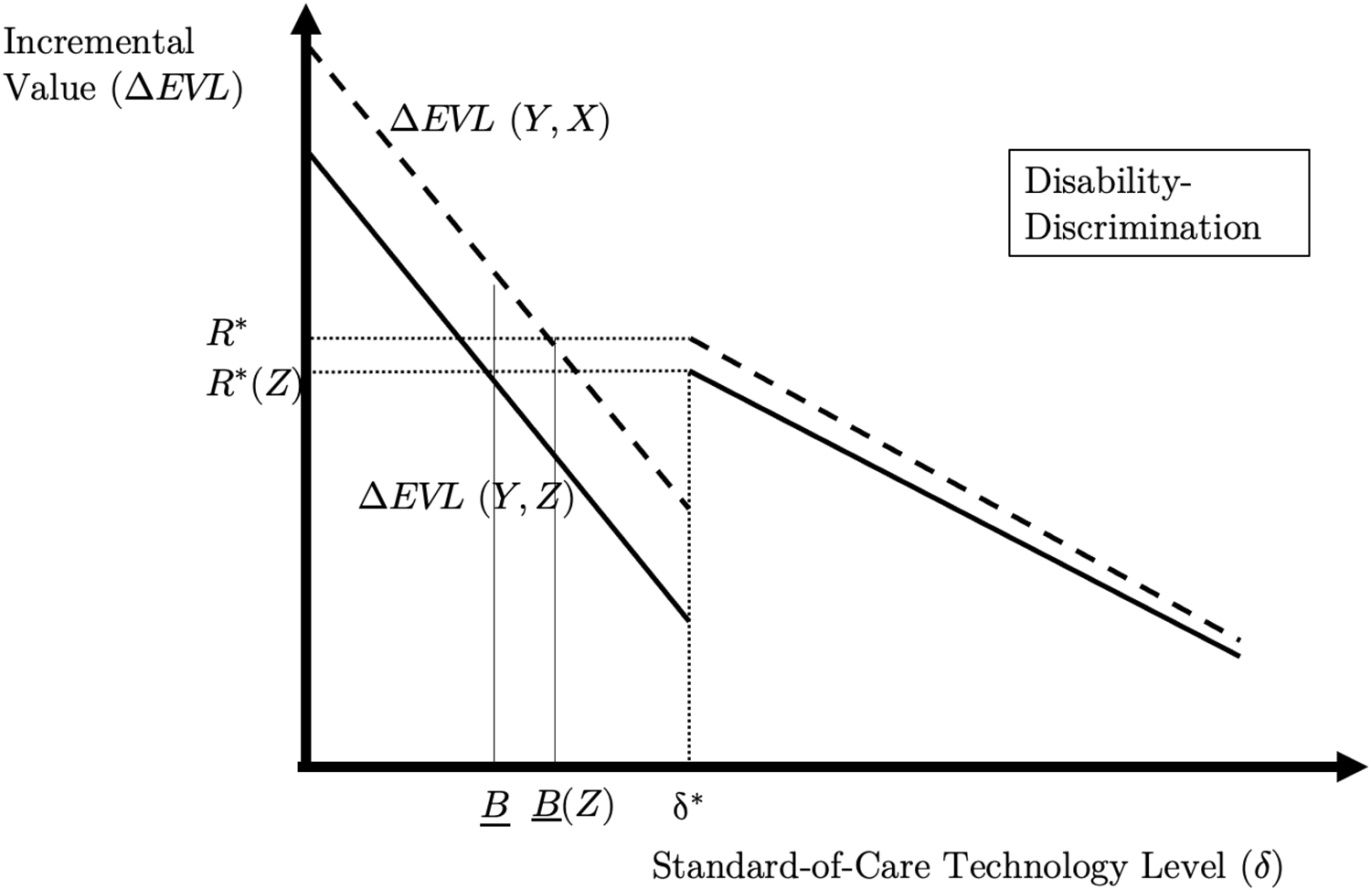
The case of disability-discrimination under EVL and HYT. Notes: The figure illustrates the relationship between $\delta$ and $\Delta {\text{EVL}}\left( {Y,X\left( \delta \right)} \right)$ for different values of $\delta$, under the case where $R^{*} > L^{*}$. The figure plots the incremental value of technology $Y$ compared to standard-of-care $X\left( \delta \right)$ for different values of $\delta$ (solid lines), and the incremental value of $Y$ compared to standard-of-care $Z\left( \delta \right)$ for different values of $\delta$ (dark dashed lines). $Z$ produces higher quality-of-life than $X$ at a single time, $t^{\prime}$, but is otherwise identical to $X$. An analogous figure obtains for $\Delta {\text{HYT}}\left( {Y,X\left( \delta \right)} \right)$ and $\Delta {\text{HYT}}\left( {Y,Z\left( \delta \right)} \right)$, but where $R_{{{\text{HYT}}}}^{*} < L_{{{\text{HYT}}}}^{*}$

The proof appears in the appendix, but Fig. 3 illustrates the intuition. The incremental value of *Y* relative to $Z$ [i.e., $\Delta {\text{EVL}}\left( {Y,Z} \right)$ or $\Delta {\text{HYT}}\left( {Y,Z} \right)$] lies below the incremental value of $Y$ relative to $X$, because $Z$ produces more quality-of-life than $X$ along with identical survival. The upward shifts narrow the width of the region, $\left( {\underline {B} ,\delta^{*} } \right)$ over which negative survival valuation originates. (The geometry is the same for both $\Delta {\text{EVL}}$ and $\Delta {\text{HYT}}$, although the points and curves on the graph may shift across the two.) As a result, there will be some standard-of-care technology levels for which survival gains harm patients with disability, but do not harm healthier patients. This creates circumstances in which life-extension is worth less (in fact, it is costly!) to those with lower quality of life.

**Fig. 4:**
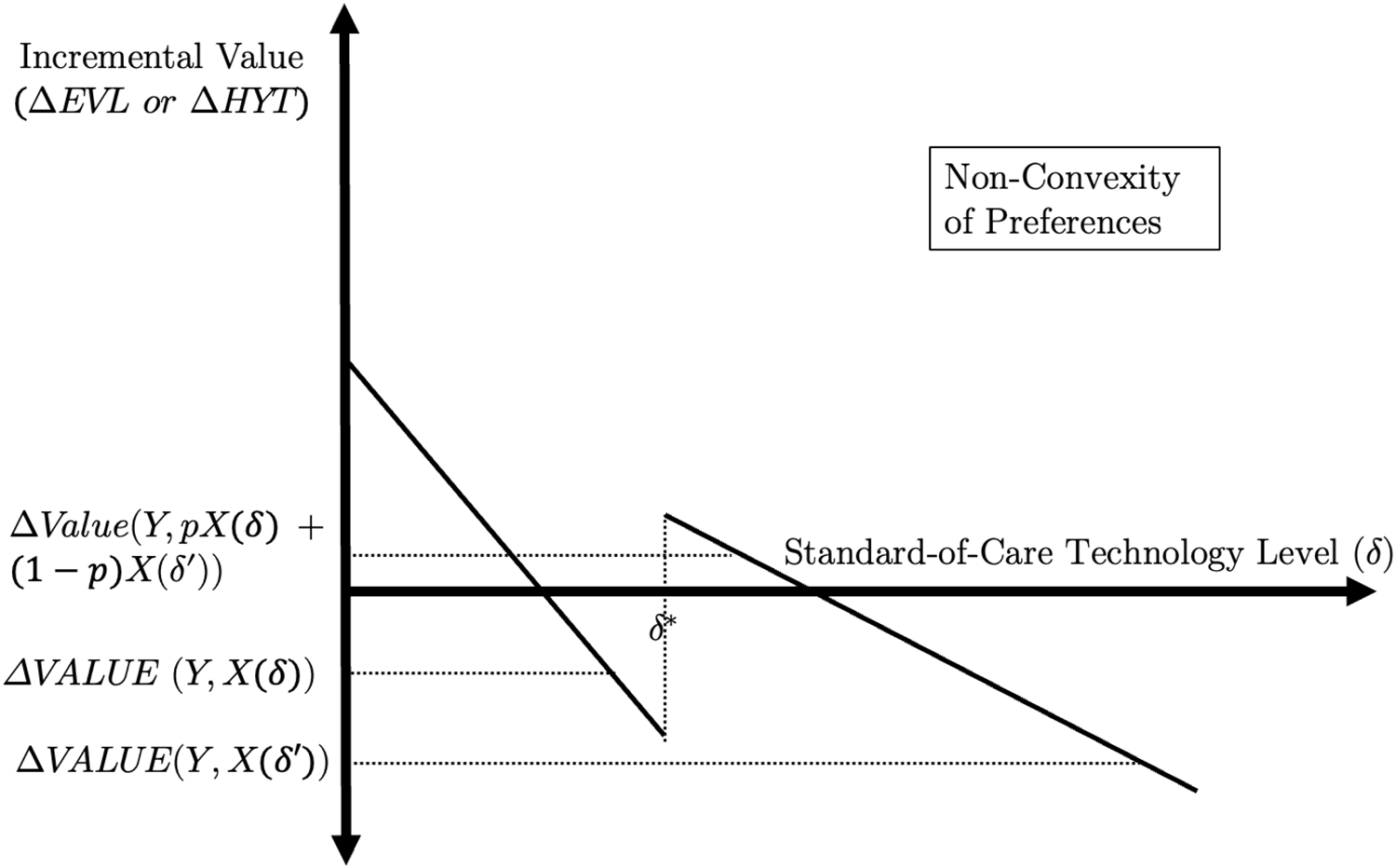
The case of non-convex preferences under EVL and HYT. Notes: The figure plots incremental values ($\Delta {\text{EVL}}\left( {Y,X\left( \delta \right)} \right)$ and $\Delta {\text{HYT}}\left( {Y,X\left( \delta \right)} \right)$ for different values of $\delta$. Incremental quality-of-life is held constant throughout the figure

*Non-convexity of survival preferences under EVL and HYT* The non-monotonicity of EVL in total survival also permits non-convex preferences over survival. Consider prior and current standard-of-care technologies $A$ and $B$, and a novel technology, $C$, such that $\Delta {\text{EVL}}\left( {C,A} \right) < 0$ and $\Delta {\text{EVL}}\left( {C,B} \right) < 0$. Under EVL and HYT, it is possible that $\Delta {\text{EVL}}\left( {C,pA + \left( {1 - p} \right)B} \right) > 0$, even though both $A$ and $B$ dominate $C$ individually. Figure 4 illustrates the intuition. Pick $\delta < \delta^{*}$ and $\delta^{\prime} > \delta^{*} ,$ such that the novel technology, $Y$, is dominated by the prior and current standard-of-care interventions $X\left( \delta \right)$ and $X\left( {\delta^{\prime}} \right)$. As long as $R_{{{\text{EVL}}}}^{*}$ lies above zero, there will be some standard-of-care intervention that lies between $X\left( \delta \right)$ and $X\left( {\delta^{\prime}} \right)$ but is dominated by the novel technology. This reveals the convex combination of $\delta$ and $\delta ^{\prime}$, such that $\Delta {\text{EVL}}\left( {Y,pX\left( \delta \right) + \left( {1 - p} \right)X\left( {\delta^{\prime}} \right)} \right) > 0$, and an analogous result will obtain for HYT. The following corollary, proven in the appendix, formalizes the intuition depicted by the figure.

##### Corollary 2.2

(Non-convex survival preferences). *Suppose the conditions of* Theorem 2*continue to hold. It then follows that the Principle of Convexity Over Survival Preferences* (*Principle* 4) *fails to hold, in the following sense*.

A) *Suppose that* $R_{{{\text{EVL}}}}^{*} > 0 > L_{{{\text{EVL}}}}^{*}$. *Under these conditions, there is a novel technology*, $Y$, *and standard-of-care technologies*, $X\left( {\delta^{\prime}} \right)$ *and* $X\left( {\delta^{\prime\prime}} \right)$, *where* $\delta^{\prime} < \delta^{*}$ *and* $\delta^{\prime\prime} > \delta^{*}$, *such that* $\Delta {\text{EVL}}\left( {Y,X\left( {\delta^{\prime}} \right)} \right) < 0$ *and* $\Delta {\text{EVL}}\left( {Y,X\left( {\delta^{\prime\prime}} \right)} \right) < 0$, *but* $\exists p \in \left( {0,1} \right)$, *such that* $\Delta {\text{EVL}}\left( {Y,pX\left( {\delta^{\prime}} \right) + \left( {1 - p} \right)X\left( {\delta^{\prime\prime}} \right)} \right) > 0$.
B) *Suppose further that* $R_{{{\text{HYT}}}}^{*} > 0 > L_{{{\text{HYT}}}}^{*}$. *Under these conditions, there is a novel technology*, $Y^{\prime}$, *and standard-of-care technologies*, $X^{\prime}\left( {\nu^{\prime}} \right)$ *and* $X^{\prime}\left( {\nu^{\prime\prime}} \right)$, *where* $\nu^{\prime} < \delta^{*}$ *and* $\nu^{\prime\prime} > \delta^{*}$, *such that* $\Delta {\text{HYT}}\left( {Y^{\prime},X^{\prime}\left( {\nu^{\prime}} \right)} \right) < 0$ *and* $\Delta {\text{HYT}}\left( {Y^{\prime},X^{\prime}\left( {\nu^{\prime\prime}} \right)} \right) < 0$, *but* $\exists p^{\prime} \in \left( {0,1} \right)$, *such that* $\Delta {\text{HYT}}\left( {Y^{\prime},p^{\prime}X^{\prime}\left( {\nu^{\prime}} \right) + \left( {1 - p^{\prime}} \right)X^{\prime}\left( {\nu^{\prime\prime}} \right)} \right) > 0$.

**Fig. 5:**
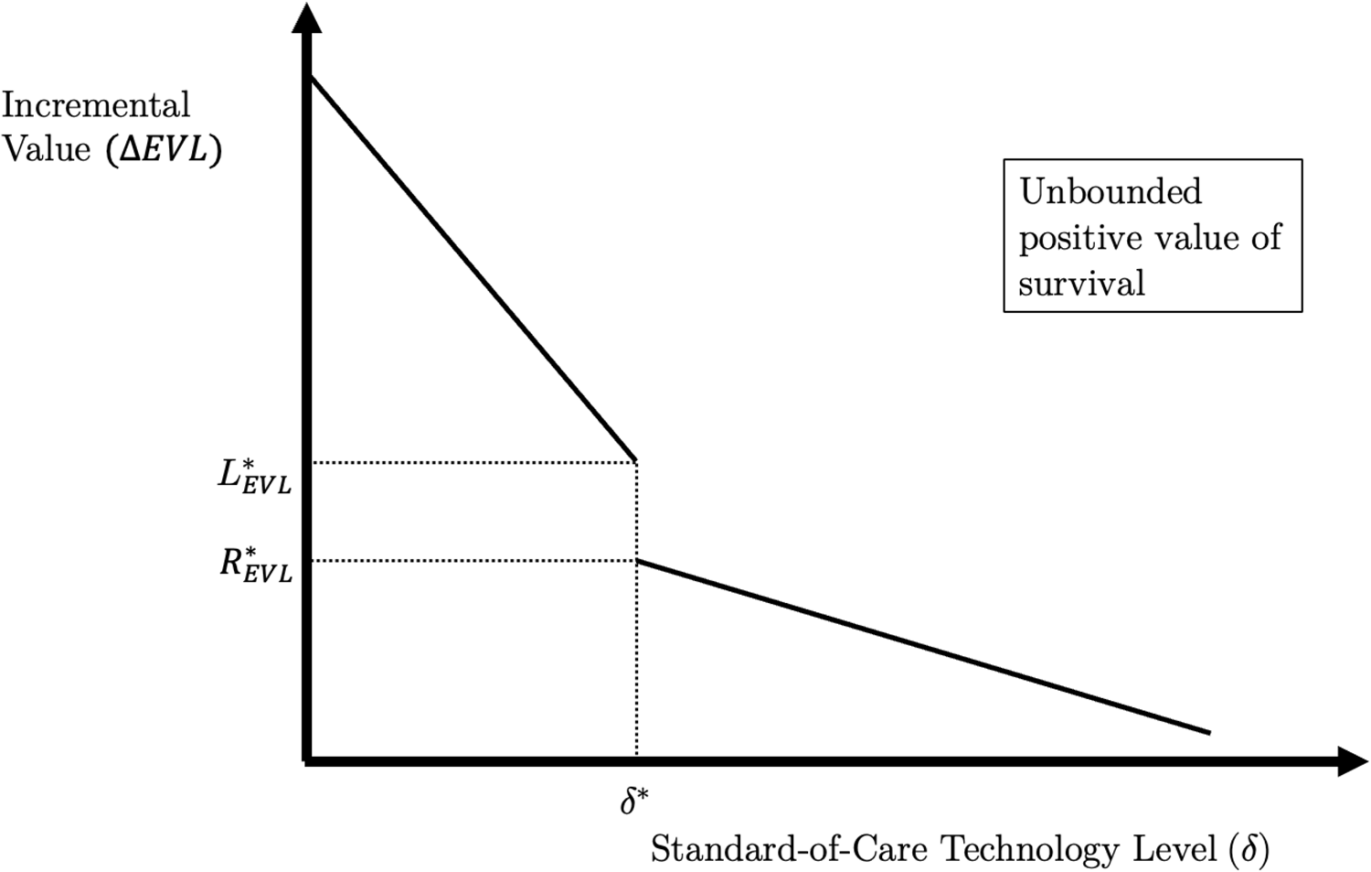
The case of unbounded positive value of survival from EVL and HYT. Notes: The figure plots incremental value for $\Delta {\text{EVL}}\left( {Y,X\left( \delta \right)} \right)$ for different values of $\delta$. Incremental quality-of-life is held constant throughout the figure. An analogous figure obtains for $\Delta {\text{HYT}}\left( {Y,X\left( \delta \right)} \right)$, but where $R_{{{\text{HYT}}}}^{*} > L_{{{\text{HYT}}}}^{*}$

*Unbounded average value of survival under EVL and HYT* The two prior anomalies arise when EVL and HYT discontinuously increase at ${\delta }^{*}$. Figure 5 illustrates what happens under the alternative case where they discontinuously decrease at $\delta^{*}$. At the discontinuity point, a zero or near-zero increase in standard-of-care survival triggers a discrete drop in both the incremental EVL and HYT metrics. Therefore, the average value of survival gains, which is the ratio between the change in incremental value and the increase in total survival, can diverge to infinity. Intuitively, there remains a discrete change in value, even though the difference in survival approaches zero; this causes the ratio between the change in value and the change in survival to diverge. This violates Principle (3), of bounded average value from survival gains.

The average value per life-year of a change in survival from $\delta^{a}$ to $\delta^{b} > \delta^{a}$ is $\frac{{\left( {\Delta {\text{Value}}\left( {Y,X\left( {\delta^{a} } \right)} \right) - \Delta {\text{Value}}\left( {Y,X\left( {\delta^{b} } \right)} \right)} \right)}}{{S\left( {\delta^{b} } \right) - S(\delta^{a} )}}$, i.e., the absolute value of the slope of the incremental value function (either $\Delta {\text{EVL}}$ or $\Delta {\text{HYT}}$) between $\delta^{a}$ and $\delta^{b}$. (Since the value function may not be differentiable everywhere, we rely on the average slope between two points instead of a derivative.) This average value of a life-year can become unbounded near the discontinuity, as formalized in the following theorem.

##### Theorem 3

(Unbounded value of a life-year) *Suppose that* $Y$ *is a novel intervention with survival and quality-of-life profiles* $\left\{ {S_{Yt} ;Q_{Yt} } \right\}_{t = 1}^{T}$, *and* $X\left( \alpha \right)$ *is a family of standard-of-care interventions with profiles* $\left\{ {S_{Xt} \alpha ;Q_{Xt} } \right\}_{t = 1}^{T}$. *The terms*, $\delta^{*}$, $\underline {B}_{{{\text{EVL}}}}$, $\overline{B}_{{{\text{EVL}}}}$, $\underline {B}_{{{\text{HYT}}}}$, and $\overline{B}_{{{\text{HYT}}}}$ *are as defined in the text*. $\Delta {\text{EVL}}$ *and* $\Delta {\text{HYT}}$ *violate Principle* (3), *in the following sense*.

A) *Suppose* $\sum\nolimits_{t} {\left( {S_{Yt} - S_{Xt} \delta^{*} } \right)Q_{Yt} < 0}$, *so that* $R_{{{\text{EVL}}}}^{*} < L_{{{\text{EVL}}}}^{*}$. *For any* $V > 0$, $\exists \delta^{\prime} \in \left( {0,\delta^{*} } \right)$ *and* $\delta^{\prime\prime} \in \left( {\delta^{*} ,1} \right)$, *such that* $\frac{{\left( {\Delta {\text{EVL}}\left( {Y,X\left( {\delta^{\prime}} \right)} \right) - \Delta {\text{EVL}}\left( {Y,X\left( {\delta^{\prime\prime}} \right)} \right)} \right)}}{{\mathop \sum \nolimits_{t} S_{{X\left( {\delta ^{\prime\prime}} \right)t}} - \mathop \sum \nolimits_{t} S_{{X\left( {\delta ^{\prime}} \right)t}} }} > V$.
B) *In contrast, suppose* $\sum\nolimits_{t} {\left( {S_{Yt} - S_{Xt} \delta^{*} } \right)Q_{Xt} < 0}$, *so that* $R_{{{\text{HYT}}}}^{*} < L_{{{\text{HYT}}}}^{*}$. *For any* $V > 0$, $\exists \delta^{\prime} \in \left( {0,\delta^{*} } \right)$ *and* $\delta^{\prime\prime} \in \left( {\delta^{*} ,1} \right)$, *such that* $\frac{{\left( {\Delta {\text{HYT}}\left( {Y,X\left( {\delta^{\prime}} \right)} \right) - \Delta {\text{HYT}}\left( {Y,X\left( {\delta^{\prime\prime}} \right)} \right)} \right)}}{{\mathop \sum \nolimits_{t} S_{{X\left( {\delta ^{\prime\prime}} \right)t}} - \mathop \sum \nolimits_{t} S_{{X\left( {\delta ^{\prime}} \right)t}} }} > V$.

Theorem (3) demonstrates that incremental EVL and HYT violate Principle (3). The proof appears in the Appendix.

### The need for discontinuity in EVL and HYT

At this point, one might ask whether the choice-inconsistencies above could be addressed by resorting to a continuous version of incremental EVL or HYT. Unfortunately, this is not viable, because continuity within the existing approaches to EVL and HYT creates other inconsistencies.

Consider the following two alternative and non-piecewise modifications to EVL, both of which would preserve continuity in survival:

17 $$\Delta {\text{Metric}}^{\prime}\left( {A,B} \right) = \mathop \sum \limits_{t} \left( {S_{At} - S_{Bt} } \right) + \mathop \sum \limits_{t} S_{Bt} \left( {Q_{At} - Q_{Bt} } \right)$$

18 $$\Delta {\text{Metric}}^{\prime\prime}\left( {A,B} \right) = \mathop \sum \limits_{t} \left( {S_{At} - S_{Bt} } \right) + \mathop \sum \limits_{t} S_{At} \left( {Q_{At} - Q_{Bt} } \right).$$

In other words, suppose we use either the standard-of-care survival profile or the novel treatment survival profile, regardless of which has more total survival. Even though they remove the discontinuity in survival, these metrics would create instability in choice, depending on which therapy is chosen as the standard of care.

#### Theorem 4

(The need for discontinuity in EVL and HYT) *Suppose there are two interventions*, *A* *and* *B*, *with sequences of quality-of-life and survival*, $\left\{ {S_{At} ;Q_{At} } \right\}_{t = 0}^{t = T}$ *and* $\left\{ {S_{Bt} ;Q_{Bt} } \right\}_{t = 0}^{t = T}$. *And suppose* $\Delta {\text{Metric}}^{\prime}$ *and* $\Delta Metric^{\prime\prime}$ *are as defined in Eqs*. (17) and (18), *respectively. Suppose that the following two conditions hold*:

19 $$\mathop \sum \limits_{t} S_{Bt} \le \mathop \sum \limits_{t} S_{At}$$

20 $$\mathop \sum \limits_{t} \left( {S_{Bt} } \right)\left( {Q_{At} - Q_{Bt} } \right) > \mathop \sum \limits_{t} \left( {S_{At} } \right)\left( {Q_{At} - Q_{Bt} } \right) > 0.$$

Under these circumstances, if $\Delta {\text{Metric}}^{\prime}\left( {B,A} \right) > 0$, then $\Delta {\text{Metric}}^{\prime}\left( {A,B} \right) > 0$, and if $\Delta {\text{Metric}}^{\prime\prime}\left( {B,A} \right) > 0$, then $\Delta {\text{Metric}}^{\prime\prime}\left( {A,B} \right) > 0$.

The theorem demonstrates that “solving” the discontinuity within incremental EVL or HYT leads to other fundamental choice-inconsistencies.

One might also wonder whether the choice-inconsistencies arise solely from the reliance on QALYs in some cases. This is not the case either. Consider an alternate formulation of incremental EVL that uses the same formula, regardless of whether the novel therapy is life-extending over standard of care

21 $$\Delta {\text{EVL}}^{\prime}\left( {Y,X} \right) = Q_{0} \mathop \sum \limits_{t} \left( {S_{Yt} - S_{Xt} } \right) + \left\{ {\begin{array}{*{20}c} {\mathop \sum \limits_{t} S_{Xt} \left( {Q_{Yt} - Q_{Xt} } \right),} & {{\text{if }}\mathop \sum \limits_{t} S_{Xt} < \mathop \sum \limits_{t} S_{Yt} } \\ {\mathop \sum \limits_{t} S_{Yt} \left( {Q_{Yt} - Q_{Xt} } \right),} & {{\text{if }}\mathop \sum \limits_{t} S_{Yt} \le \mathop \sum \limits_{t} S_{Xt} } \\ \end{array} } \right..$$

One can imagine a similar reconstruction of incremental HYT as in

22 $$\Delta {\text{HYT}}\left( {Y,X} \right) = \Delta {\text{EVL}}\left( {Y,X} \right) + \left\{ {\begin{array}{*{20}c} {\mathop \sum \limits_{t} \left( {S_{Yt} - S_{Xt} } \right)\left( {Q_{Yt} - Q_{Xt} } \right),} & {{\text{if }}\mathop \sum \limits_{t} S_{Xt} < \mathop \sum \limits_{t} S_{Yt} } \\ {\mathop \sum \limits_{t} \left( {S_{Xt} - S_{Yt} } \right)\left( {Q_{Yt} - Q_{Xt} } \right),} & {{\text{if }}\mathop \sum \limits_{t} S_{Yt} \le \mathop \sum \limits_{t} S_{Xt} } \\ \end{array} } \right..$$

Unfortunately, these modifications remain subject to the same violations, because there continues to be a discontinuity in total survival at the point where the formula switches from using standard-of-care survival profiles to novel therapy survival profiles. In particular, Theorem 1, Corollary 1.1, Theorem 2, Corollary 2.2, and Theorem 3 would all continue to hold.^11^

## A consistent choice-theoretic approach to eliminating discrimination

### The principle-consistency of GRACE

EVL and HYT result in violations of key principles for decision-making. Nonetheless, both EVL and HYT emerged to address a well-documented empirical phenomenon: the apparent willingness of consumers and third-party payers to pay more to treat severe illness. A choice-consistent alternative is still needed that satisfies the Principle of Non-Discrimination Against the Sick and Disabled (Principle 5) and, correspondingly, the requirements of US law.

Neither EVL nor HYT have been shown to result from a well-defined utility function. As a result, they do not inherit the stable choice-theoretic properties of neoclassical microeconomics. In contrast, Generalized Risk-Adjusted Cost-Effectiveness (GRACE)—along with any other metric based on a neoclassical expected utility-maximization framework—readily satisfies Principles (1), (2), (3), and (4), and Condition (1). The following theorem sets forth the weak conditions required for this to be true.

#### Theorem 5:

(Principle-consistency of GRACE and QALY metrics) *Define the metrics*, $\Delta {\text{QALY}}$ *and* $\Delta {\text{GRACE}}$ *as specified in Eqs*. (1) and (4), *respectively. Let* $W$ *be any continuously differentiable and weakly monotonic HRQoL utility function defined over the domain*, $Q \in \left[ {0,1} \right]$, *where* $W\left( Q \right) \ge 0$ *and* $W\left( 0 \right) = 0$. *For a given pre-existing quality-of-life level bounded away from zero in the sense that* $\exists \underline{{Q_{0} }} \in \left( {0,1} \right)$, *such that* $Q_{0} \le \underline{{Q_{0} }} < 0$, *both* $\Delta {\text{QALY}}$ *and* $\Delta {\text{GRACE}}$ *satisfy Principles* (1), (2), (3), *and* (4), *along with Condition* (1).

The proof is straightforward and appears in the appendix. Note that Theorem 5 does not yet address the principle of non-discrimination (Principle 5). It is well understood that QALYs violate Principle (5). GRACE’s consistency with this principle remains in question, since prior research suggests GRACE can be consistent with equity under certain conditions [1, 19]. We turn to this issue next.

Under GRACE, the marginal value of life-extension is $\frac{{U\left( c \right)W\left( {Q_{T} } \right)}}{{U^{\prime}\left( c \right)W\left( {Q_{0} } \right)}}$. $U$ and $W$ measure utility over consumption and health-related quality of life, respectively. Non-medical consumption is $c$. $Q_{T}$ is the quality-of-life level in the post-treatment state, and $Q_{0}$ is the quality-of-life level in the pre-treatment state. The numerator in this expression is the marginal utility of an additional period of life under the new treatment, and the denominator is the marginal utility of consumption in the ex ante pre-illness state. As noted earlier, pre-existing disability and/or illness have countervailing effects on the value of life-extension under GRACE; however, constant relative risk-aversion utility ensures that the value of life-extension does not change with pre-existing disability under GRACE. Under GRACE, the value of life-extension equals utility in the post-treatment state divided by utility in the pre-illness state. Thus, when that ratio remains constant, so does the value of life-extension. With the CRRA assumption, utility is proportional to quality-of-life. Let us assume further that disability reduces quality-of-life proportionally, in both the pre-illness and post-treatment states. With these two assumptions, disability will lower pre-illness utility by the same percentage as it lowers post-treatment utility. This leaves the ratio between the two unchanged and, with it, the value of life-extension.

To characterize these conditions more formally, consider patients in “high” pre-existing health (Type $H$) and patients in “low” pre-existing health (Type $L$). It facilitates the analysis to measure health loss as proportional decline from perfect health. Therefore, define $Q_{P}$ as the quality-of-life for a consumer in perfect health. Define $d^{*}$ as the proportional HRQoL burden of permanent disability and pre-existing illness suffered by a given patient, such that pre-illness quality-of-life is given by $Q_{0} = \left( {1 - d^{*} } \right)Q_{P}$. Define $d_{H}^{*} \ge 0$ as the disability burden for Type $H$ patients and define $d_{L}^{*} > 0$ as the disability burden for Type $L$ patients. We assume Type $L$ patients face higher pre-existing disability burdens, so that $d_{L}^{*} > d_{H}^{*}$.

Without loss of generality, consider a single post-treatment period, $t = 1$. Define $Q_{T \cdot H}$ and $Q_{T \cdot L}$ as post-treatment quality-of-life for Type $H$ and Type $L$ patients. Define $t_{H}^{*}$ and $t_{L}^{*}$ as the post-treatment quality-of-life burden of the acute illness among Type $H$ and Type $L$ patients, respectively, so that $Q_{T \cdot H} = \left( {1 - t_{H}^{*} } \right)Q_{P}$ and $Q_{T \cdot L} = \left( {1 - t_{L}^{*} } \right)Q_{P}$. Finally, for comparison purposes, define $Q_{T}$ as post-treatment quality-of-life for patients in perfect pre-existing health, and $t^{*}$ as the corresponding post-treatment quality-of-life burden, where $Q_{T} = \left( {1 - t^{*} } \right)Q_{P} .$ With these definitions in hand, the following theorem characterizes the conditions under which GRACE complies with the principle of equity for the sick and disabled.

#### Theorem 6

(Non-discriminatory value assessment with GRACE) *If utility over HRQoL*, $W\left( Q \right)$*,* *exhibits constant relative risk-aversion, and if* $\left( {1 - t_{j}^{*} } \right) = \left( {1 - d_{j}^{*} } \right)\left( {1 - t^{*} } \right)$ *for all Type* $j$ *patients, then GRACE complies with Principle* (5), *non-discrimination against the disabled and sick*.

The proof appears in the appendix. Mechanically, the value of life-extension for Type $j$ patients is given by $\frac{{W\left( {\left( {1 - t_{j}^{*} } \right)Q_{P} } \right)}}{{W\left( {\left( {1 - d_{j}^{*} } \right)Q_{P} } \right)}}$. Under CRRA utility, $W$ varies proportionately with quality-of-life. Moreover, if $\left( {1 - t_{j}^{*} } \right) = \left( {1 - d_{j}^{*} } \right)\left( {1 - t^{*} } \right)$, pre-existing disability lowers both pre-existing and post-treatment health by the same proportion. Therefore, pre-existing disability does not affect the ratio, $\frac{{W\left( {\left( {1 - t_{j}^{*} } \right)Q_{P} } \right)}}{{W\left( {\left( {1 - d_{j}^{*} } \right)Q_{P} } \right)}}$, and it thus fails to affect the value of life-extension.

### A worked example comparing EVL, HYT, and GRACE

To illustrate the implementation of non-discriminatory GRACE, we offer a simple example. Consider a two-period setting, where the first period is the baseline pre-illness period and the second period takes place after the illness occurs. A standard-of-care technology produces survival probabilities of 1.0 and 0.1 in the first and second periods, respectively, while a novel intervention produces survival probabilities of 1.0 and 1.0. Now, suppose these technologies are used on a population without prior disability, with quality-of-life equal to 1.0 and 0.8 in the first and second periods, and also on a population with prior disability, with quality-of-life equal to 0.5 and 0.4 in the first and second periods. Notice how the quality-of-life assumptions comply with the conditions of Theorem 6: the onset of illness proportionally lowers quality-of-life by 20%, for both the disabled and non-disabled patients. Table 1 summarizes the assumptions. Since these interventions only increase survival, non-discrimination requires that their value be the same across disability status.

**Table 1:** Comparing QALYs to non-discriminatory metrics of value

	Without prior disability		With prior disability	
	*T* = 0	*T* = 1	*T* = 0	*T* = 1
Standard of care				
Survival	1	0.1	1	0.1
QoL	1	0.8	0.5	0.4
Intervention				
Survival	1	1	1	1
QoL	1	0.8	0.5	0.4
Value of intervention				
ΔQALYs	0.72		0.36	
ΔEVL	0.90		0.90	
ΔHYT	0.90		0.90	
ΔGRACE	0.77		0.77	

Notes: GRA-QALYs are calculated assuming a health-related QoL utility function that exhibits constant relative risk-aversion with risk-aversion parameter 0.2822

The incremental QALYs produced by the intervention are given by $$\left( {1.0 - 0.1} \right)*0.8 = 0.72$$ for the non-disabled, but $$\left( {1.0 - 0.1} \right)*0.4 = 0.36$$ for the disabled; this reflects the discriminatory nature of QALYs. In contrast, the incremental equal value of life-years gained does not vary with disability status. For the non-disabled, $$\Delta {\text{EVL}} = \left( {1 - 0.1} \right) + 0.1*\left( {0.8 - 0.8} \right) = 0.9$$, and for the disabled, $$\Delta {\text{EVL}} = \left( {1 - 0.1} \right) + 0.1*\left( {0.4 - 0.4} \right) = 0.9$$. Since there are no quality-of-life improvements, $$\Delta {\text{HYT}}$$ is identical to $$\Delta {\text{EVL}}$$, where $$\Delta {\text{HYT}} = \left( {1 - 0.1} \right) + 1*\left( {0.8 - 0.8} \right) = 0.9$$ for the non-disabled and $$\Delta {\text{HYT}} = \left( {1 - 0.1} \right) + 1*\left( {0.4 - 0.4} \right) = 0.9$$ for the disabled. In this example, GRACE is implemented using a utility function over health-related quality of life that exhibits constant relative risk-aversion with a risk-aversion parameter of 0.2822 [30]. Specifically, this implies the utility function, $$W\left( H \right) = \frac{{H^{1 - \rho } }}{1 - \rho }$$, where$$\rho = 0.2822$$, the coefficient of relative risk-aversion. In this simple setting, $$\Delta {\text{GRACE}}\left( {Y,X;Q_{0} } \right) = S_{Y1} \frac{{W\left( {Q_{Y1} } \right)}}{{W\left( {Q_{0} } \right)}} - S_{X1} \frac{{W\left( {Q_{X1} } \right)}}{{W\left( {Q_{0} } \right)}}$$. Under CRRA utility, this becomes, $$\Delta {\text{GRACE}}\left( {Y,X;Q_{0} } \right) = S_{Y1} \left( {\frac{{Q_{Y1} }}{{Q_{0} }}} \right)^{{\left( {1 - \rho } \right)}} - S_{X1} \left( {\frac{{Q_{X1} }}{{Q_{0} }}} \right)^{{\left( {1 - \rho } \right)}}$$. Recall that GRACE will avoid discrimination so long as disability leaves $$\left( {\frac{{Q_{Y1} }}{{Q_{0} }}} \right)$$ and $$\left( {\frac{{Q_{X1} }}{{Q_{0} }}} \right)$$ unchanged. For the non-disabled, $$Q_{0} = 1$$, $$Q_{Y1} = Q_{X1} = 0.8$$, and $$\frac{{Q_{Y1} }}{{Q_{0} }} = \frac{{Q_{X1} }}{{Q_{0} }} = 0.8$$. For the disabled, $$Q_{0} = 0.5$$, $$Q_{Y1} = Q_{X1} = 0.4$$, and $$\frac{{Q_{Y1} }}{{Q_{0} }} = \frac{{Q_{X1} }}{{Q_{0} }} = \frac{0.4}{{0.5}} = 
0.8$$. Therefore, the ratio of post-illness to pre-illness quality-of-life is always 0.8, regardless of disability status. For both groups, $$S_{Y1} = 1.0$$ and $$S_{X1} = 0.1$$. Computation reveals that:

$$\Delta {\text{GRACE}}\left( {Y,X;Q_{0} = 1} \right) = 1.0\left( {0.8} \right)^{0.7178} - 0.1\left( {0.8} \right)^{0.7178} = 0.77$$

$$\Delta {\text{GRACE}}\left( {Y,X;Q_{0} = 0.5} \right) = 1.0\left( {0.8} \right)^{0.7178} - 0.1\left( {0.8} \right)^{0.7178} = 0.77.$$

Therefore, GRACE values life-extension equally under the conditions of Theorem 6.

The mechanics of this example reveals some important implications for practice. If disability had additive, rather than multiplicative, effects on quality-of-life, the conditions of Theorem 6 would have been violated. For instance, suppose that disability reduced quality-of-life by 0.2 units, both before and after the onset of illness. In such a case, the disabled population would exhibit quality-of-life equal to 0.8 before illness and 0.6 post-illness. Hence, $\frac{{Q_{Y1} }}{{Q_{0} }} = 0.8$ for the non-disabled, but $\frac{{Q_{Y1} }}{{Q_{0} }} = \frac{0.6}{{0.8}} = 0.75$ for the disabled. Since the ratio of post-illness to pre-illness quality-of-life varies with disability, GRACE does not avoid disability-discrimination in this case, even though CRRA utility holds. The assumption of proportional quality-of-life reductions from disability is needed.

Alternatively, consider the importance of the CRRA assumption. Imagine constant absolute risk-aversion (CARA) utility, as embodied in $W\left( H \right) = \frac{{1 - e^{ - H} }}{{1 - \frac{1}{e}}}$. In this case, the coefficient of relative risk-aversion is proportional to $H$. Computation reveals that $\frac{{W\left( {Q_{Y1} } \right)}}{{W\left( {Q_{0} } \right)}} = \frac{{1 - e^{{ - Q_{Y1} }} }}{{1 - e^{{ - Q_{0} }} }}$ and that dividing both $Q_{Y1}$ and $Q_{0}$ by half would reduce $\frac{{W\left( {Q_{Y1} } \right)}}{{W\left( {Q_{0} } \right)}}$ rather than leaving it unaffected. Below, we discuss perspectives on the empirical validity or invalidity of the CRRA utility assumption.

## Conclusion

Traditional cost-effectiveness fails to align with the apparent disease severity premium exhibited by consumers and payers. It also fails to conform with recent changes to US federal law prohibiting metrics that discriminate against the disabled, aged, and terminally ill. Ad hoc approaches to addressing these limitations of traditional CEA introduce choice pathologies that render them inappropriate for policymaking. GRACE offers a choice-consistent alternative that admits a premium for disease severity and avoids prohibited discrimination under well-defined conditions on utility and the effects of disability.

The beguiling pursuit of a health utility measure that affords a maximum health state to all persons receiving life-extension has led us to models that can fail to satisfy a set of nearly self-evident principles for choice. Its lack of microeconomic or choice-theoretic foundations ultimately compromises the EVL approach. By building upon EVL, HYT inherits these underlying inconsistencies. Rather than making ad hoc adjustments to the QALY, GRACE avoids discrimination by restricting risk posture in a neoclassical economic model of consumer behavior.

In contrast to EVL or HYT, GRACE states its conditions in terms of empirically observable preferences and measured health, within a choice-consistent microeconomic framework. Its vulnerability to discriminatory outcomes lies in the possibility that empirical preferences, or health, may not conform with legislated restrictions or collective preferences for non-discrimination. In other words, the required restrictions on utility, or on the quality-of-life effects of disability, may be rejected by the data. This raises the possibility that non-discriminatory value assessment under GRACE may not always be externally consistent with data in some patient populations. Ideally, CRRA would prove to be an empirical reasonable assumption in populations of interest, and in any event, the assumption ought to be tested before relying on this form of the utility function. However, even if CRRA fails to be the best-fitting utility function empirically, GRACE remains internally consistent, because it is built upon standard microeconomic tools. Practitioners of cost-effectiveness tolerate external inconsistency in several forms. For instance, economic assessments almost never incorporate real-world variation in per capita consumption that would otherwise imply that fewer medical resources ought to be allocated to the poor than to the rich. Even more on point, EVL and HYT themselves embrace external inconsistency in the measurement of quality-of-life. Complete external consistency is impossible to achieve, or nearly so, when relying on analytical models, all of which abstract away from key features of the real-world. It is up to the analyst to weigh the importance of empirical accuracy against societal considerations like equity. However, consistency with widely held principles of non-discriminatory choice—additivity, monotonicity and boundedness in the value of survival, convexity in survival, and non-discrimination itself—ought to remain an inviolable requirement of healthcare decision analysis.

## Mathematical appendix

### Theorem 1

(Additive incremental value). *There exist health interventions* $A$, $B$, $C$, *and* $D$, *such that* $\Delta {\text{EVL}}\left( {A,B} \right) > \Delta {\text{EVL}}\left( {C,D} \right)$, but $\Delta {\text{EVL}}\left( {A,C} \right) < \Delta {\text{EVL}}\left( {B,D} \right)$. There exist interventions $A$, $B$, $C$, and $D$, such that $\Delta {\text{HYT}}\left( {A,B} \right) > \Delta {\text{HYT}}\left( {C,D} \right)$ but $\Delta {\text{HYT}}\left( {A,C} \right) < \Delta {\text{HYT}}\left( {B,D} \right)$. Therefore, $\Delta {\text{EVL}}$ and $\Delta {\text{HYT}}$ violate Principle (1).

### Proof of Theorem 1

#### Proof for $\Delta {\text{EVL}}$

Suppose there are three time periods. In period 1, $D$ is the standard-of-care technology, while $B$ and $C$ are novel technologies. In period 2, $C$ is the only standard-of-care technology, while $A$ and $B$ are novel technologies. In period 3, $B$ is the only standard-of-care technology, while $A$ is a novel technology. Denote $\Delta {\text{EVL}}^{i} \left( {X,Y} \right)$ as incremental EVL estimated in period $i$ over technologies $X$ and $Y$. Suppose also $\mathop \sum \limits_{t} S_{At} > \mathop \sum \limits_{t} S_{Bt} > \mathop \sum \limits_{t} S_{Ct} > \mathop \sum \limits_{t} S_{Dt}$.

Now, suppose further that

23 $$\mathop \sum \limits_{t} S_{At} - \mathop \sum \limits_{t} S_{Bt} { } < \mathop \sum \limits_{t} S_{Ct} - \mathop \sum \limits_{t} S_{Dt} .$$

This inequality implies

24 $$\mathop \sum \limits_{t} S_{At} - \mathop \sum \limits_{t} S_{Ct} { } < { }\mathop \sum \limits_{t} S_{Bt} - \mathop \sum \limits_{t} S_{Dt} .$$

Assuming (23), implicitly define $\epsilon > 0$ such that

25 $$\mathop \sum \limits_{t} S_{At} - \mathop \sum \limits_{t} S_{Bt} + { }\varepsilon = \mathop \sum \limits_{t} S_{Ct} - \mathop \sum \limits_{t} S_{Dt} .$$

Whenever the following condition is met, Principle (1) will fail for EVL:

26 $$\left[ {\mathop \sum \limits_{t} S_{Bt} Q_{At} - \mathop \sum \limits_{t} S_{Bt} Q_{Bt} } \right] - \left[ {\mathop \sum \limits_{t} S_{Dt} Q_{Ct} - \mathop \sum \limits_{t} S_{Dt} Q_{Dt} } \right] > \epsilon > \left[ {\mathop \sum \limits_{t} S_{Ct} Q_{At} - \mathop \sum \limits_{t} S_{Ct} Q_{Ct} } \right] - \left[ {\mathop \sum \limits_{t} S_{Dt} Q_{Bt} - \mathop \sum \limits_{t} S_{Dt} Q_{Dt} } \right].$$

To see why, observe that the left-hand inequality in expression (26), combined with Eq. (25), implies

27 $$\left[ {\mathop \sum \limits_{t} S_{At} - \mathop \sum \limits_{t} S_{Bt} } \right] + \left[ {\mathop \sum \limits_{t} S_{Bt} Q_{At} - \mathop \sum \limits_{t} S_{Bt} Q_{Bt} } \right] - \left[ {\mathop \sum \limits_{t} S_{Dt} Q_{Ct} - \mathop \sum \limits_{t} S_{Dt} Q_{Dt} } \right] > \mathop \sum \limits_{t} S_{Ct} - \mathop \sum \limits_{t} S_{Dt} .$$

This is equivalent to $\Delta {\text{EVL}}^{3} \left( {A,B} \right) > \Delta {\text{EVL}}^{1} \left( {C,D} \right)$. However, the right-hand inequality in expression (26), combined with Eq. (25), implies

28 $$\mathop \sum \limits_{t} S_{At} - \mathop \sum \limits_{t} S_{Bt} + \left[ {\mathop \sum \limits_{t} S_{Ct} Q_{At} - \mathop \sum \limits_{t} S_{Ct} Q_{Ct} } \right] - \left[ {\mathop \sum \limits_{t} S_{Dt} Q_{Bt} - \mathop \sum \limits_{t} S_{Dt} Q_{Dt} } \right] < \mathop \sum \limits_{t} S_{Ct} - \mathop \sum \limits_{t} S_{Dt} .$$

This is equivalent to $\Delta {\text{EVL}}^{2} \left( {A,C} \right) < \Delta {\text{EVL}}^{1} \left( {B,D} \right)$.

#### Proof for $\Delta {\text{HYT}}$

Suppose there are three time periods. In period 1, $D$ is the standard-of-care technology, while $C$ is a novel technology. In period 2, $C$ and *D* are standard-of-care technologies, while $B$ is a novel technology. In period 3, $B$ and $C$ are the only standard-of-care technologies, while $A$ is a novel technology. In period 4, $B$ is the only standard-of-care technology, and $A$ is a novel technology. Denote $\Delta {\text{HYT}}^{i} \left( {X,Y} \right)$ as incremental HYT estimated in period $i$ over technologies $X$ and $Y$. Suppose also $\mathop \sum \limits_{t} S_{At} > \mathop \sum \limits_{t} S_{Bt} > \mathop \sum \limits_{t} S_{Ct} > \mathop \sum \limits_{t} S_{Dt}$.

Analogously, the condition below will imply that Principle (1) will fail for HYT

29 $$\left[ {\mathop \sum \limits_{t} S_{At} Q_{At} - \mathop \sum \limits_{t} S_{At} Q_{Bt} } \right] - \left[ {\mathop \sum \limits_{t} S_{Ct} Q_{Ct} - \mathop \sum \limits_{t} S_{Ct} Q_{Dt} } \right] > \epsilon > \left[ {\mathop \sum \limits_{t} S_{At} Q_{At} - \mathop \sum \limits_{t} S_{At} Q_{Ct} } \right] - \left[ {\mathop \sum \limits_{t} S_{Bt} Q_{Bt} - \mathop \sum \limits_{t} S_{Bt} Q_{Dt} } \right].$$

The left-hand inequality in expression (29), combined with Eq. (25), implies

30 $$\mathop \sum \limits_{t} S_{At} - \mathop \sum \limits_{t} S_{Bt} + \left[ {\mathop \sum \limits_{t} S_{At} Q_{At} - \mathop \sum \limits_{t} S_{At} Q_{Bt} } \right] - \left[ {\mathop \sum \limits_{t} S_{Ct} Q_{Ct} - \mathop \sum \limits_{t} S_{Ct} Q_{Dt} } \right] > \mathop \sum \limits_{t} S_{Ct} - \mathop \sum \limits_{t} S_{Dt} .$$

This is equivalent to $\Delta {\text{HYT}}^{4} \left( {A,B} \right) > \Delta {\text{HYT}}^{1} \left( {C,D} \right)$. However, the right-hand inequality in expression (29), combined with Eq. (25), implies

31 $$\mathop \sum \limits_{t} S_{At} - \mathop \sum \limits_{t} S_{Bt} + \left[ {\mathop \sum \limits_{t} S_{At} Q_{At} - \mathop \sum \limits_{t} S_{At} Q_{Ct} } \right] - \left[ {\mathop \sum \limits_{t} S_{Bt} Q_{Bt} - \mathop \sum \limits_{t} S_{Bt} Q_{Dt} } \right] < \mathop \sum \limits_{t} S_{Ct} - \mathop \sum \limits_{t} S_{Dt} .$$

This is equivalent to $\Delta {\text{HYT}}^{3} \left( {A,C} \right) < \Delta {\text{HYT}}^{2} \left( {B,D} \right)$.

Finally, we confirm that the intersection between the set of interventions satisfying $\sum\nolimits_{t} {S_{At} } > \sum\nolimits_{t} {S_{Bt} } > \sum\nolimits_{t} {S_{Ct} } > \sum\nolimits_{t} {S_{Dt} }$, expression (26), and expression (29) is nonempty. The following set of interventions satisfies all these constraints: $S_{A} = \left\{ {0.99,0.99,0.99} \right\}$, $Q_{A} = \left\{ {0.9,1.0,0.01} \right\}$, $S_{B} = \left\{ {0.9,0.9,0.9} \right\}$, $Q_{B} = \left\{ {0.02,0.01,1.0} \right\}$, $S_{C} = \left\{ {0.4,0.4,0.4} \right\}$, $Q_{C} = \left\{ {1.0,0.8,0.01} \right\}$, $S_{D} = \left\{ {0.2,0.2,0.2} \right\}$, and $Q_{D} = \left\{ {0.01,0.01,1.0} \right\}$. Computation reveals the following:

$$\epsilon = 0.33$$

$$\left[ {\mathop \sum \limits_{t} S_{Bt} Q_{At} - \mathop \sum \limits_{t} S_{Bt} Q_{Bt} } \right] - \left[ {\mathop \sum \limits_{t} S_{Dt} Q_{Ct} - \mathop \sum \limits_{t} S_{Dt} Q_{Dt} } \right] = 0.634$$

$$\left[ {\mathop \sum \limits_{t} S_{Ct} Q_{At} - \mathop \sum \limits_{t} S_{Ct} Q_{Ct} } \right] - \left[ {\mathop \sum \limits_{t} S_{Dt} Q_{Bt} - \mathop \sum \limits_{t} S_{Dt} Q_{Dt} } \right] = 0.038$$

$$\left[ {\mathop \sum \limits_{t} S_{At} Q_{At} - \mathop \sum \limits_{t} S_{At} Q_{Bt} } \right] - \left[ {\mathop \sum \limits_{t} S_{Ct} Q_{Ct} - \mathop \sum \limits_{t} S_{Ct} Q_{Dt} } \right] = 0.5552$$

$$\left[ {\mathop \sum \limits_{t} S_{At} Q_{At} - \mathop \sum \limits_{t} S_{At} Q_{Ct} } \right] - \left[ {\mathop \sum \limits_{t} S_{Bt} Q_{Bt} - \mathop \sum \limits_{t} S_{Bt} Q_{Dt} } \right] = 0.09.$$

QED

##### Corollary 1.1

(Failure of indirect comparison). *There exist health interventions* $A$, $B$, *and* $C$, *such that* $\Delta {\text{EVL}}\left( {A,C} \right) > \Delta {\text{EVL}}\left( {B,C} \right)$ *but* $\Delta {\text{EVL}}\left( {A,B} \right) < 0$. *There exist interventions* $A$,$B$, *and* $C$, *such that* $\Delta {\text{HYT}}\left( {A,C} \right) > \Delta {\text{HYT}}\left( {B,C} \right)$ *but* $\Delta {\text{HYT}}\left( {A,B} \right) < 0$. *Therefore*, $\Delta {\text{EVL}}$ *and* $\Delta {\text{HYT}}$ *violate Condition* (1).

### Proof of Corollary 1.1

#### Proof for ${\Delta }EVL$

The proof of the corollary follows the same outline as that of Theorem 1. Suppose there are two time periods. In period 1, $C$ is the standard-of-care technology, while $A$ and $B$ are novel technologies. In period 2, $B$ is accepted as the sole standard-of-care technology, while $A$ remains a novel technology. Suppose $\sum\nolimits_{t} {S_{At} } > \sum\nolimits_{t} {S_{Bt} } > \sum\nolimits_{t} {S_{Ct} }$. This implies that

32 $$\mathop \sum \limits_{t} S_{At} - \mathop \sum \limits_{t} S_{Ct} > { }\mathop \sum \limits_{t} S_{Bt} - \mathop \sum \limits_{t} S_{Ct} .$$

Assuming expression (32), implicitly define $\epsilon$, such that

33 $$\mathop \sum \limits_{t} S_{At} = \mathop \sum \limits_{t} S_{Bt} + \epsilon.$$

Whenever the following condition is met, Principle (1) will fail for EVL:

34 $$\mathop \sum \limits_{{\text{t}}} S_{Bt} \left( {Q_{Bt} - Q_{At} } \right) > \epsilon > \mathop \sum \limits_{{\text{t}}} S_{Ct} \left( {Q_{Bt} - Q_{At} } \right).$$

The right-hand inequality in expression (34), combined with Eq. (33), implies

35 $$\mathop \sum \limits_{t} \left( {S_{At} - S_{Ct} } \right) + \mathop \sum \limits_{{\text{t}}} S_{Ct} \left( {Q_{At} - Q_{Ct} } \right) > \mathop \sum \limits_{t} \left( {S_{Bt} - S_{Ct} } \right) + \mathop \sum \limits_{{\text{t}}} S_{Ct} \left( {Q_{Bt} - Q_{Ct} } \right).$$

This demonstrates that $\Delta {\text{EVL}}^{1} \left( {A,C} \right) > \Delta {\text{EVL}}^{1} \left( {B,C} \right)$. However, the left-hand inequality in expression (34), combined with Eq. (33), implies

36 $$\mathop \sum \limits_{t} S_{At} - \mathop \sum \limits_{t} S_{Bt} + \mathop \sum \limits_{{\text{t}}} S_{Bt} \left( {Q_{At} - Q_{Bt} } \right) < 0.$$

This demonstrates that $\Delta {\text{EVL}}^{2} \left( {A,B} \right) < 0$, which proves the result.

#### Proof for $\Delta {\text{HYT}}$

Continue with the same technologies as above but adjust the timing. Suppose there are two time periods. In period 1, $C$ is the standard-of-care technology, while $B$ is a novel technology. In period 2, $B$ joins $C$ as a standard-of-care technology, and a new technology, $A$, emerges as a novel alternative. Suppose $\sum\nolimits_{t} {S_{At} } > \sum\nolimits_{t} {S_{Bt} } > \sum\nolimits_{t} {S_{Ct} }$.

Now, whenever the following condition is met, Principle (1) will fail for HYT:

37 $$\mathop \sum \limits_{{\text{t}}} S_{At} \left( {Q_{Bt} - Q_{At} } \right) > \epsilon > \mathop \sum \limits_{{\text{t}}} \left( {S_{Bt} Q_{Bt} - S_{At} Q_{At} } \right) + \mathop \sum \limits_{{\text{t}}} \left( {S_{At} Q_{Ct} - S_{Bt} Q_{Ct} } \right).$$

The left-hand inequality in expression (37), combined with Eq. (33), implies

38 $$\mathop \sum \limits_{t} S_{At} - \mathop \sum \limits_{t} S_{Bt} + \mathop \sum \limits_{{\text{t}}} S_{At} \left( {Q_{At} - Q_{Bt} } \right) < 0.$$

This demonstrates that $\Delta {\text{HYT}}^{2} \left( {A,B} \right) < 0$. However, the right-hand inequality in expression (37), combined with Eq. (33), implies

39 $$\mathop \sum \limits_{t} \left( {S_{At} - S_{Ct} } \right) + \mathop \sum \limits_{{\text{t}}} S_{At} \left( {Q_{At} - Q_{Ct} } \right) > \mathop \sum \limits_{t} \left( {S_{Bt} - S_{Ct} } \right) + \mathop \sum \limits_{{\text{t}}} S_{Bt} \left( {Q_{Bt} - Q_{Ct} } \right).$$

This demonstrates that $\Delta {\text{HYT}}^{2} \left( {A,C} \right) > \Delta {\text{HYT}}^{1} \left( {B,C} \right)$, proving the violation.

Finally, the intersection between the set of interventions satisfying $\sum\nolimits_{t} {S_{At} } > \sum\nolimits_{t} {S_{Bt} } > \sum\nolimits_{t} {S_{Ct} }$, expression (34), and expression (37) is nonempty. The following set of interventions satisfies all these constraints: $S_{A} = \left\{ {0.99,0.99,0.99} \right\}$, $Q_{A} = \left\{ {0.7,0.5,1.0} \right\}$, $S_{B} = \left\{ {0.9,0.9,0.9} \right\}$, $Q_{B} = \left\{ {0.7,0.9,1} \right\}$, $S_{C} = \left\{ {0.1,0.1,0.1} \right\}$, and $Q_{C} = \left\{ {0.2,0.2,0.01} \right\}$. Computation reveals the following:

$$\epsilon = 0.27$$

$$\mathop \sum \limits_{{\text{t}}} S_{Bt} \left( {Q_{Bt} - Q_{At} } \right) = 0.36$$

$$\mathop \sum \limits_{{\text{t}}} S_{Ct} \left( {Q_{Bt} - Q_{At} } \right) = 0.04$$

$$\mathop \sum \limits_{{\text{t}}} S_{At} \left( {Q_{Bt} - Q_{At} } \right) = 0.396$$

$$\mathop \sum \limits_{t} \left( {S_{Bt} - S_{Ct} } \right) + \mathop \sum \limits_{{\text{t}}} S_{Bt} \left( {Q_{Bt} - Q_{Ct} } \right) = 0.1989.$$

QED

##### Theorem 2

(Negative value of survival gains) *Suppose that* $T \ge 2$, $Y$ *is a novel intervention with survival and quality-of-life profiles* $\left\{ {S_{Yt} ;Q_{Yt} } \right\}_{t = 1}^{T}$, *and* $X\left( \delta \right)$ *is the family of standard-of-care interventions with profile* $\left\{ {S_{Xt} \delta ;Q_{Xt} } \right\}_{t = 1}^{T}$ *where* $\sum\nolimits_{t} {S_{Yt} } < \sum\nolimits_{t} {S_{Xt} }$, *and* $0 \le \delta \le 1$ *is an index of technological progress*.

A) *Under these conditions, there exist standard-of-care interventions* $X\left( \phi \right)$ *and* $X\left( {\phi^{\prime}} \right)$, *such that* $X\left( {\phi^{\prime}} \right)$ *features strictly higher survival and weakly higher quality of life than* $X\left( \phi \right)$, *but that* $\Delta {\text{EVL}}\left( {Y,X\left( {\phi^{\prime}} \right)} \right) > \Delta {\text{EVL}}\left( {Y,X\left( \phi \right)} \right)$.
B) *There also exist standard-of-care interventions* $X\left( \rho \right)$ *and* $X\left( {\rho^{\prime}} \right)$, *such that* $X\left( {\rho^{\prime}} \right)$ *features strictly higher survival and weakly higher quality of life than* $X\left( \rho \right)$, *but that* $\Delta {\text{HYT}}\left( {Y,X\left( {\rho^{\prime}} \right)} \right) > \Delta {\text{HYT}}\left( {Y,X\left( \rho \right)} \right)$.

### Proof of Theorem 2

The theorem can be restated as follows. Suppose that $T \ge 2$, $Y$ is a novel intervention with strictly positive survival and quality-of-life profile $\left\{ {S_{Yt} ;Q_{Yt} } \right\}_{t = 1}^{T}$, and $X\left( \delta \right)$ is the family of standard-of-care interventions with strictly positive survival and quality-of-life profiles$\left\{ {S_{Xt} \delta ;Q_{Xt} } \right\}_{t = 1}^{T}$, where $0 \le \delta \le 1$, and where $\sum\nolimits_{t} {S_{Xt} } > \sum\nolimits_{t} {S_{Yt} }$. The terms, $\delta^{*}$, $\underline {B}_{{{\text{EVL}}}}$, $\overline{B}_{{{\text{EVL}}}}$, $\underline {B}_{{{\text{HYT}}}}$, and $\overline{B}_{{{\text{HYT}}}}$ are as defined in the text. Under these conditions: A) if $\sum\nolimits_{t} {\left( {S_{Yt} - S_{Xt} \delta^{*} } \right)Q_{Yt} > 0}$, then, $\forall \phi \in \left( {\underline {B}_{{{\text{EVL}}}} ,\delta^{*} } \right)$, $\exists \phi^{\prime} \in \left( {\delta^{*} ,\overline{B}_{{{\text{EVL}}}} } \right)$, such that$\Delta {\text{EVL}}\left( {Y,X\left( {\phi^{\prime}} \right)} \right) > \Delta {\text{EVL}}\left( {Y,X\left( \phi \right)} \right)$; and B) if $\sum\nolimits_{t} {\left( {S_{Yt} - S_{Xt} \delta^{*} } \right)Q_{Xt} > 0}$, then, $\forall \rho \in \left( {\underline {B}_{{{\text{HYT}}}} ,\delta^{*} } \right)$, $\exists \rho^{\prime} \in \left( {\delta^{*} ,\overline{B}_{{{\text{HYT}}}} } \right)$, such that $\Delta {\text{HYT}}\left( {Y,X\left( {\rho^{\prime}} \right)} \right) > \Delta {\text{HYT}}\left( {Y,X\left( \rho \right)} \right)$.

We will begin by proving the result for $\Delta {\text{EVL}}$ and will then turn to $\Delta {\text{HYT}}$.

#### Proof for $\Delta {\text{EVL}}$

For any $\delta \in \left( {0,\delta^{*} } \right)$, $\Delta {\text{EVL}}\left( {Y,X\left( \delta \right)} \right) = \sum\nolimits_{t} {\left( {S_{Yt} - S_{Xt} \delta } \right)} + \sum\nolimits_{t} {S_{Xt} \delta \left( {Q_{Yt} - Q_{Xt} } \right)}$, and for any $\delta \in \left( {\delta^{*} ,1} \right)$, $\Delta{\text{EVL}}\left( {Y,X\left( \delta \right)} \right) = \sum\nolimits_{t} {\left( {S_{Yt} Q_{Yt} - S_{Xt} \delta Q_{Xt} } \right)}$. Both these functions are clearly continuous over the regions specified, and differentiation readily confirms they are also both monotonically decreasing in $\delta$ over the regions specified. Under the conditions specified by Part A of the theorem, $\sum\nolimits_{t} {\left( {S_{Yt} - S_{Xt} \delta^{*} } \right)Q_{Yt} > 0}$. This implies $L_{{{\text{EVL}}}}^{*} < R_{{{\text{EVL}}}}^{*}$. Pick some $\phi \in \left( {\underline {B}_{{{\text{EVL}}}} ,\delta^{*} } \right)$. Since $\Delta {\text{EVL}}\left( {Y,X\left( {\underline {B}_{{{\text{EVL}}}} } \right)} \right) = R_{{{\text{EVL}}}}^{*}$, $\lim_{{\delta \to \delta^{* - } }} \Delta {\text{EVL}}\left( {Y,X\left( \delta \right)} \right) = L_{{{\text{EVL}}}}^{*}$, $\Delta {\text{EVL}}$ is continuous and monotonic decreasing for all $0 < \delta < \delta^{*}$, and $\phi \in \left( {\underline {B}_{{{\text{EVL}}}} ,\delta^{*} } \right)$, it follows that $L_{{{\text{EVL}}}}^{*} < \Delta {\text{EVL}}\left( {Y,X\left( \phi \right)} \right) < R_{{{\text{EVL}}}}^{*}$. Since $\Delta {\text{EVL}}\left( {Y,X\left( {\overline{B}_{{{\text{EVL}}}} } \right)} \right) = L_{{{\text{EVL}}}}^{*}$, $\lim_{{\delta \to \delta^{* + } }} \Delta {\text{EVL}}\left( {Y,X\left( \delta \right)} \right) = R_{{{\text{EVL}}}}^{*}$, and $\Delta {\text{EVL}}$ is continuous over the interval $\left( {\delta^{*} ,\overline{B}_{{{\text{EVL}}}} } \right)$, the intermediate-value theorem implies that $\forall W \in \left( {R_{{{\text{EVL}}}}^{*} ,L_{{{\text{EVL}}}}^{*} } \right)$, there exists some $\phi^{\prime} \in \left( {\delta^{*} ,\overline{B}_{{{\text{EVL}}}} } \right)$, such that $\Delta{\text{EVL}}\left( {Y,X\left( {\phi^{\prime}} \right)} \right) = W$. Since $L_{{{\text{EVL}}}}^{*} < {\text{EVL}}\left( {Y,X\left( \phi \right)} \right) < R_{{{\text{EVL}}}}^{*}$, it follows that $R_{{{\text{EVL}}}}^{*} - \frac{{R_{{{\text{EVL}}}}^{*} - \Delta {\text{EVL}}\left( {Y,X\left( \phi \right)} \right)}}{2} \in \left( {R_{{{\text{EVL}}}}^{*} ,L_{{{\text{EVL}}}}^{*} } \right)$. Therefore, the intermediate-value theorem implies that $\exists \phi^{\prime} \in \left( {\delta^{*} ,\overline{B}_{{{\text{EVL}}}} } \right)$, such that $\Delta {\text{EVL}}\left( {Y,X\left( {\phi^{\prime}} \right)} \right) = R_{{{\text{EVL}}}}^{*} - \frac{{R_{{{\text{EVL}}}}^{*} - \Delta {\text{EVL}}\left( {Y,X\left( \phi \right)} \right)}}{2} > \Delta {\text{EVL}}\left( {Y,X\left( \phi \right)} \right)$. *QED.*

#### Proof for $\Delta {\text{HYT}}$

For any $\delta \in \left( {0,\delta^{*} } \right)$, $\Delta {\text{HYT}}\left( {Y,X\left( \delta \right)} \right) = \sum\nolimits_{t} {\left( {S_{Yt} - S_{Xt} \delta } \right)} + \sum\nolimits_{t} {S_{Yt} \left( {Q_{Yt} - Q_{Xt} } \right)}$, and for any $\delta \in \left( {\delta^{*} ,1} \right)$, $\Delta {\text{HYT}}\left( {Y,X\left( \delta \right)} \right) = \sum\nolimits_{t} {\left( {S_{Yt} Q_{Yt} - S_{Xt} \delta Q_{Xt} } \right)}$. Both these functions are clearly continuous over the regions specified, and differentiation readily confirms they are also both monotonic decreasing in $\delta$. Under the conditions specified by Part B of the theorem, $\sum\nolimits_{t} {\left( {S_{Yt} - S_{Xt} \delta^{*} } \right)Q_{Xt} > 0}$. This implies that $L_{{{\text{HYT}}}}^{*} < R_{{{\text{HYT}}}}^{*}$. Pick some $\rho \in \left( {\underline {B}_{{{\text{HYT}}}} ,\delta^{*} } \right)$. Since $\Delta {\text{HYT}}\left( {Y,X\left( {\underline {B}_{{{\text{HYT}}}} } \right)} \right) = R_{{{\text{HYT}}}}^{*}$, $\lim_{{\delta \to \delta^{* - } }} \Delta {\text{HYT}}\left( {Y,X\left( \delta \right)} \right) = L_{{{\text{HYT}}}}^{*}$, $\Delta {\text{HYT}}$ is continuous and monotonic decreasing for all $0 < \delta < \delta^{*}$, and $\rho \in \left( {\underline {B}_{{{\text{HYT}}}} ,\delta^{*} } \right)$, it follows that $L_{{{\text{HYT}}}}^{*} < \Delta {\text{HYT}}\left( {Y,X\left( \rho \right)} \right) < R_{{{\text{HYT}}}}^{*}$. Since $\Delta {\text{HYT}}\left( {Y,X\left( {\overline{B}_{{{\text{HYT}}}} } \right)} \right) = L_{{{\text{HYT}}}}^{*}$, $\lim_{{\delta \to \delta^{* + } }} \Delta {\text{HYT}}\left( {Y,X\left( \delta \right)} \right) = R_{{{\text{HYT}}}}^{*}$, and $\Delta {\text{HYT}}$ is continuous over the interval $\left( {\delta^{*} ,\overline{B}_{{{\text{HYT}}}} } \right)$, the intermediate-value theorem implies that $\forall W \in \left( {R_{{{\text{HYT}}}}^{*} ,L_{{{\text{HYT}}}}^{*} } \right)$, there exists some $\rho^{\prime} \in \left( {\delta^{*} ,\overline{B}_{{{\text{HYT}}}} } \right)$, such that $\Delta {\text{HYT}}\left( {Y,X\left( {\rho^{\prime}} \right)} \right) = W$. Since $L_{{{\text{HYT}}}}^{*} < \Delta {\text{HYT}}\left( {Y,X\left( \rho \right)} \right) < R_{{{\text{HYT}}}}^{*}$, it follows that $R_{{{\text{HYT}}}}^{*} - \frac{{R_{{{\text{HYT}}}}^{*} - \Delta {\text{HYT}}\left( {Y,X\left( \rho \right)} \right)}}{2} \in \left( {R_{{{\text{HYT}}}}^{*} ,L_{{{\text{HYT}}}}^{*} } \right)$. Therefore, the intermediate-value theorem implies $\exists \rho^{\prime} \in (\delta^{*} ,\overline{B}_{{{\text{HYT}}}} )$, such that $\Delta {\text{HYT}}\left( {Y,X\left( {\rho^{\prime}} \right)} \right) = R_{{{\text{HYT}}}}^{*} - \frac{{R_{{{\text{HYT}}}}^{*} - \Delta {\text{HYT}}\left( {Y,X\left( \rho \right)} \right)}}{2} > \Delta {\text{HYT}}\left( {Y,X\left( \rho \right)} \right)$. *QED.*

##### Corollary 2.1:

(Disability-discrimination). *Assume that* *Y* *and* $X\left( \delta \right)$ *are defined as described in the conditions of* Theorem 2, *and suppose* $\exists {{t^{\prime}}}$, *such that* $\frac{{S_{{Xt^{\prime}}} }}{{\mathop \sum \nolimits_{t} S_{Xt} }} > \frac{{S_{{Yt^{\prime}}} }}{{\mathop \sum \nolimits_{t} S_{Yt} }}$ *and* $Q_{{Xt^{\prime}}} \in \left( {0,1} \right)$. *In this context, Condition* (2) *is violated by both EVL and HYT, in the following way*.

A) *When computing incremental EVL for technologies* $X$ *and* $Y$, *suppose* $Y$ *produces higher survival than* $X$ *in at least one time period. Under these conditions, there exists an intervention*, $Z$, *producing the same survival as* $X$ *but strictly higher quality-of-life than* $X$, *and* $0 < \phi^{\prime\prime} < \phi^{\prime\prime\prime} < 1$, *where* $\Delta {\text{EVL}}\left( {Y,Z\left( {\phi^{\prime\prime}} \right)} \right) - \Delta {\text{EVL}}\left( {Y,Z\left( {\phi^{\prime\prime\prime}} \right)} \right) > 0,$ *but* $\Delta {\text{EVL}}\left( {Y,X\left( {\phi^{\prime\prime}} \right)} \right) - \Delta {\text{EVL}}\left( {Y,X\left( {\phi^{\prime\prime\prime}} \right)} \right) < 0$.
B) *Alternatively, when computing incremental HYT for technologies* $X$ *and* $Y$, *suppose* $Y$ *produces lower survival than* $X$ *in at least one time period. Under these conditions, there exists an intervention*, $Z$, *producing the same survival as* $X$ *but strictly higher quality-of-life than* $X$, *and* $0 < \phi^{\prime\prime} < \phi^{\prime\prime\prime} < 1$, *where* $\Delta {\text{HYT}}\left( {Y,Z\left( {\phi^{\prime\prime}} \right)} \right) - \Delta {\text{HYT}}\left( {Y,Z\left( {\phi^{\prime\prime\prime}} \right)} \right) > 0$ *but* $\Delta {\text{HYT}}\left( {Y,X\left( {\phi^{\prime\prime}} \right)} \right) - \Delta {\text{HYT}}\left( {Y,X\left( {\phi^{\prime\prime\prime}} \right)} \right) < 0.$

### Proof of Corollary 2.1

The corollary can be restated as follows. Suppose the conditions of Theorem 2 continue to hold and suppose further that: (a) when computing incremental EVL for standard-of care *X* and novel intervention *Y*, $\exists {{t^{\prime}}},$ such that $1 \le t^{\prime} \le T$, $S_{{Xt^{\prime}}} > S_{{Yt^{\prime}}}$, and $Q_{{Xt^{\prime}}} \in \left( {0,1} \right)$; and (b) when computing incremental HYT for standard-of-care *X* and novel intervention *Y*, $\exists {{t^{\prime}}},$ such that $\frac{{S_{{Xt^{\prime}}} }}{{\mathop \sum \nolimits_{t} S_{Xt} }} > \frac{{S_{{Yt^{\prime}}} }}{{\mathop \sum \nolimits_{t} S_{Yt} }}$ and $Q_{{Xt^{\prime}}} \in \left( {0,1} \right)$.

A) Under these conditions, there exists for incremental EVL an intervention, $Z$, with $1 \ge Q_{{Zt^{\prime}}} > Q_{{Xt^{\prime}}}$ but otherwise identical to $X$, $\phi^{\prime\prime} \in \left( {\underline {B}_{{{\text{EVL}}}} ,\delta^{*} } \right)$, and $\phi^{\prime\prime\prime} \in \left( {\delta^{*} ,\overline{B}_{{{\text{EVL}}}} } \right)$, such that $\Delta {\text{EVL}}\left( {Y,Z\left( {\phi^{\prime\prime}} \right)} \right) - \Delta {\text{EVL}}\left( {Y,Z\left( {\phi^{\prime\prime\prime}} \right)} \right) > 0$ but $\Delta {\text{EVL}}\left( {Y,X\left( {\phi^{\prime\prime}} \right)} \right) - \Delta {\text{EVL}}\left( {Y,X\left( {\phi^{\prime\prime\prime}} \right)} \right) < 0$.
B) Separately, under these conditions, there exists for incremental HYT an intervention, $Z$, with $1 \ge Q_{{Zt^{\prime}}} > Q_{{Xt^{\prime}}}$ but otherwise identical to $X$, $\rho^{\prime\prime} \in \left( {\underline {B}_{{{\text{HYT}}}} ,\delta^{*} } \right)$, and $\rho^{\prime\prime\prime} \in \left( {\delta^{*} ,\overline{B}_{{{\text{HYT}}}} } \right)$, such that $\Delta {\text{HYT}}\left( {Y,Z\left( {\rho^{\prime\prime}} \right)} \right) - \Delta {\text{HYT}}\left( {Y,Z\left( {\rho^{\prime\prime\prime}} \right)} \right) > 0$ but $\Delta {\text{HYT}}\left( {Y,X\left( {\rho^{\prime\prime}} \right)} \right) - \Delta {\text{HYT}}\left( {Y,X\left( {\rho^{\prime\prime\prime}} \right)} \right) < 0$.

#### Proof for $\Delta {\text{EVL}}$

Differentiate expression (13) with respect to $Q_{{Xt^{\prime}}}$, recognizing that $R_{{{\text{EVL}}}}^{*}$ also depends on $Q_{{Xt^{\prime}}}$, to obtain

40 $$\frac{{{\text{d}}\underline {B}_{{{\text{EVL}}}} }}{{{\text{d}}Q_{{Xt^{\prime}}} }} = \frac{{S_{{Xt^{\prime}}} \left( {\delta^{*} - \underline {B}_{{{\text{EVL}}}} } \right)}}{{\mathop \sum \nolimits_{t} S_{Xt} \left( {1 - Q_{Yt} + Q_{Xt} } \right)}} > 0.$$

Since $Q_{Xt} > 0$ for at least one value of $t$, since $S_{{Xt^{\prime}}} > S_{{Yt^{\prime}}} \ge 0$, and since $\delta^{*} > \underline {B}_{{{\text{EVL}}}}$, it follows that $\frac{{{\text{d}}\overline{B}_{{{\text{EVL}}}} }}{{{\text{d}}Q_{{Xt^{\prime}}} }} > 0$. Define the intervention, $Z$, to have identical survival and quality-of-life profiles as $X$, but with $1 \ge Q_{{Zt^{\prime}}} > Q_{{Xt^{\prime}}}$. The technology, $Z$, when compared to $X$, may feature different values of $R_{{{\text{EVL}}}}^{*}$, $\underline {B}_{{{\text{EVL}}}}$, and $\overline{B}_{{{\text{EVL}}}}$. Accordingly, therefore, define the right-hand limit, $R_{{{\text{EVL}}}}^{*} \left( Z \right)$ and lower bound, $\underline {B}_{{{\text{EVL}}}} \left( Z \right)$ associated with $Z$, implicitly as

41 $$R_{{{\text{EVL}}}}^{*} \left( {\text{Z}} \right) \equiv \mathop {\lim }\limits_{{\delta \to \delta^{* + } }} \Delta {\text{EVL}}\left( {Y,Z\left( \delta \right)} \right) = \mathop \sum \limits_{t} S_{Yt} Q_{Yt} - S_{Zt} \delta^{*} Q_{Zt}$$

42 $$\mathop \sum \limits_{t} \left( {S_{Yt} - S_{Zt} \underline {B}_{{{\text{EVL}}}} \left( Z \right)} \right) + \mathop \sum \limits_{t} S_{Zt} \underline {B}_{{{\text{EVL}}}} \left( Z \right)\left( {Q_{Yt} - Q_{Zt} } \right) = R_{{{\text{EVL}}}}^{*} \left( Z \right).$$

Notice that $\delta^{*}$ is identical between Eqs. (38) and (39), because the technology $Z$ and the technology $X$ have the same survival profile. Thus, $\delta^{*} = \frac{{\mathop \sum \nolimits_{t} S_{Yt} }}{{\mathop \sum \nolimits_{t} S_{Xt} }} = \frac{{\mathop \sum \nolimits_{t} S_{Yt} }}{{\mathop \sum \nolimits_{t} S_{Zt} }}$.

Since $Z$ involves higher quality-of-life at time $t^{\prime}$ than $Y$, it follows from Eq. (40) that $\underline {B}_{{{\text{EVL}}}} \left( Z \right) > \underline {B}_{{{\text{EVL}}}}$. Moreover, since the conditions of Theorem 2 also apply to $Z$, $R_{{{\text{EVL}}}}^{*} \left( Z \right) > L_{{{\text{EVL}}}}^{*} \left( Z \right)$. Therefore, it must be the case that $\delta^{*} > \underline {B}_{{{\text{EVL}}}} \left( Z \right).$ Pick some $\phi^{\prime\prime} \in \left( {\underline {B}_{{{\text{EVL}}}} ,\underline {B}_{{{\text{EVL}}}} \left( Z \right)} \right)$. According to Theorem 2, there exists $\phi^{\prime\prime\prime} \in \left( {\delta^{*} ,\overline{B}_{{{\text{EVL}}}} } \right)$, such that $\Delta {\text{EVL}}\left( {Y,X\left( {\phi^{\prime\prime}} \right)} \right) - \Delta {\text{EVL}}\left( {Y,X\left( {\phi^{\prime\prime\prime}} \right)} \right) < 0$*.* However, since $\phi^{\prime\prime} < \underline {B}_{{{\text{EVL}}}} \left( Z \right)$ and $\phi^{\prime\prime\prime} > \delta^{*}$, $\Delta {\text{EVL}}\left( {Y,Z\left( {\phi^{\prime\prime}} \right)} \right) > R_{{{\text{EVL}}}}^{*} \left( Z \right) > \Delta {\text{EVL}}\left( {Y,Z\left( {\phi^{\prime\prime\prime}} \right)} \right) > 0$*.* Thus, $\Delta {\text{EVL}}\left( {Y,Z\left( {\phi^{\prime\prime}} \right)} \right) - \Delta {\text{EVL}}\left( {Y,Z\left( {\phi^{\prime\prime\prime}} \right)} \right) > 0$. *QED.*

#### Proof for $\Delta {\text{HYT}}$

Differentiate expression (15) with respect to $Q_{{Yt^{\prime}}}$, recognizing that $R_{{{\text{HYT}}}}^{*}$ also depends on $Q_{{Yt^{\prime}}}$, to obtain

43 $$\frac{{{\text{d}}\underline {B}_{{{\text{HYT}}}} }}{{{\text{d}}Q_{{Xt^{\prime}}} }} = \frac{{S_{{Xt^{\prime}}} \delta^{*} - S_{{Yt^{\prime}}} }}{{\mathop \sum \nolimits_{t} S_{Xt} }} > 0.$$

Since the corollary assumes $\frac{{S_{{Xt^{\prime}}} }}{{\mathop \sum \nolimits_{t} S_{Xt} }} > \frac{{S_{{Yt^{\prime}}} }}{{\mathop \sum \nolimits_{t} S_{Yt} }}$, it implies that $S_{{Xt^{\prime}}} \delta^{*} - S_{{Yt^{\prime}}} > 0$. It thus follows that $\frac{{{\text{d}}\underline {B}_{{{\text{HYT}}}} }}{{{\text{d}}Q_{{Yt^{\prime}}} }} > 0$. Define the intervention, $Z$, to have identical survival and quality-of-life profiles as $X$, but with $1 \ge Q_{{Zt^{\prime}}} > Q_{{Xt^{\prime}}}$; the latter must be possible, because the corollary assumes $Q_{{Xt^{\prime}}} < 1$. Furthermore, define $Z$ such that $\sum\nolimits_{t} {\left( {S_{Yt} - S_{Zt} \delta^{*} } \right)Q_{Zt} > 0}$; since $\sum\nolimits_{t} {\left( {S_{Yt} - S_{Xt} \delta^{*} } \right)Q_{Xt} > 0}$ with strict equality, it must be possible to choose $Q_{{Zt^{\prime}}} > Q_{{Xt^{\prime}}}$ while still ensuring that this condition holds. In other words, as long as $Q_{{Zt^{\prime}}}$ is chosen, such that $Q_{{Zt^{\prime}}} - Q_{{Xt^{\prime}}}$ is sufficiently small, all these conditions can be satisfied.

The technology, *Z*, when compared to *X*, may feature different values of $R_{{{\text{HYT}}}}^{*}$, $\underline {B}_{{{\text{HYT}}}}$ and $\overline{B}_{{{\text{HYT}}}}$. Accordingly, therefore, define the right-hand limit, $R_{{{\text{HYT}}}}^{*} \left( Z \right)$ and lower bound, $\underline {B}_{{{\text{HYT}}}} \left( Z \right)$ associated with *Z*, implicitly as

44 $$R_{{{\text{HYT}}}}^{*} \left( Z \right) \equiv \mathop {\lim }\limits_{{\delta \to \delta^{* + } }} \Delta {\text{HYT}}\left( {Y,Z\left( \delta \right)} \right) = \mathop \sum \limits_{t} S_{Yt} Q_{Yt} - S_{Zt} \delta^{*} Q_{Zt}$$

45 $$\mathop \sum \limits_{t} \left( {S_{Yt} - S_{Zt} \left( {\underline {B}_{{{\text{HYT}}}} } \right)} \right) + \mathop \sum \limits_{t} S_{Yt} \left( {Q_{Yt} - Q_{Zt} } \right) = R_{{{\text{HYT}}}}^{*} \left( Z \right).$$

Notice that $\delta^{*}$ is identical for technology *Z* and technology $X$, because they have the same survival profiles. Thus, $\delta^{*} = \frac{{\mathop \sum \nolimits_{t} S_{Yt} }}{{\mathop \sum \nolimits_{t} S_{Xt} }} = \frac{{\mathop \sum \nolimits_{t} S_{Yt} }}{{\mathop \sum \nolimits_{t} S_{Zt} }}$.

Since *Z* involves higher quality-of-life at time $t^{\prime}$ than $X$, it follows from Eq. (43) that $\underline {B}_{{{\text{HYT}}}} \left( Z \right) > \underline {B}_{{{\text{HYT}}}}$. Moreover, since $\sum\nolimits_{t} {\left( {S_{Yt} - S_{Zt} \delta^{*} } \right)Q_{Zt} > 0}$, it follows that $R_{{{\text{HYT}}}}^{*} \left( Z \right) > L_{{{\text{HYT}}}}^{*} \left( Z \right)$. Therefore, it must be the case that $\delta^{*} > \underline {B}_{{{\text{HYT}}}} \left( Z \right)$. Pick some $\rho^{\prime\prime} \in \left( {\underline {B}_{{{\text{HYT}}}} ,\underline {B}_{{{\text{HYT}}}} \left( Z \right)} \right)$. According to Theorem 2, there exists $\rho^{\prime\prime\prime} \in \left( {\delta^{*} ,\overline{B}_{{{\text{HYT}}}} } \right)$, such that $\Delta {\text{HYT}}\left( {Y,X\left( {\rho^{\prime\prime}} \right)} \right) - \Delta {\text{HYT}}\left( {Y,X\left( {\rho^{\prime\prime\prime}} \right)} \right) < 0$. However, since $\rho^{\prime\prime} < \underline {B}_{{{\text{HYT}}}} \left( Z \right)$ and $\rho^{\prime\prime\prime} > \delta^{*}$, $\Delta {\text{HYT}}\left( {Y,Z\left( {\rho^{\prime\prime}} \right)} \right) > R_{{{\text{HYT}}}}^{*} \left( Z \right) > \Delta {\text{HYT}}\left( {Y,Z\left( {\rho^{\prime\prime\prime}} \right)} \right) > 0$. Thus, $\Delta {\text{HYT}}\left( {Y,Z\left( {\rho^{\prime\prime}} \right)} \right) - \Delta {\text{HYT}}\left( {Y,Z\left( {\rho^{\prime\prime\prime}} \right)} \right) > 0$. QED.

##### Corollary 2.2:

(Non-convex survival preferences) *Suppose the conditions of* Theorem 2*continue to hold. It then follows that the Principle of Convexity Over Survival Preferences* (*Principle* 4) *fails to hold, in the following sense*.

A) *Suppose that* $R_{{{\text{EVL}}}}^{*} > 0 > L_{{{\text{EVL}}}}^{*}$. *Under these conditions, there is a novel technology*, $Y$, *and standard-of-care technologies*, $X\left( {\delta^{\prime}} \right)$ *and* $X\left( {\delta^{\prime\prime}} \right)$, *where* $\delta^{\prime} < \delta^{*}$ *and* $\delta^{\prime\prime} > \delta^{*}$, *such that* $\Delta {\text{EVL}}\left( {Y,X\left( {\delta^{\prime}} \right)} \right) < 0$ *and* $\Delta {\text{EVL}}\left( {Y,X\left( {\delta^{\prime\prime}} \right)} \right) < 0$, *but* $\exists p \in \left( {0,1} \right)$, *such that* $\Delta {\text{EVL}}\left( {Y,pX\left( {\delta^{\prime}} \right) + \left( {1 - p} \right)X\left( {\delta^{\prime\prime}} \right)} \right) > 0$.
B) *Suppose further that* $R_{{{\text{HYT}}}}^{*} > 0 > L_{{{\text{HYT}}}}^{*}$. *Under these conditions, there is a novel technology*, $Y^{\prime}$, *and standard-of-care technologies*, $X^{\prime}\left( {\nu^{\prime}} \right)$ *and* $X^{\prime}\left( {\nu^{\prime\prime}} \right)$, *where* $\nu^{\prime} < \delta^{*}$ *and* $\nu^{\prime\prime} > \delta^{*}$, *such that* $\Delta {\text{HYT}}\left( {Y^{\prime},X^{\prime}\left( {\nu^{\prime}} \right)} \right) < 0$ *and* $\Delta {\text{HYT}}\left( {Y^{\prime},X^{\prime}\left( {\nu^{\prime\prime}} \right)} \right) < 0$, *but* $\exists p^{\prime} \in \left( {0,1} \right)$, *such that* $\Delta {\text{HYT}}\left( {Y^{\prime},p^{\prime}X^{\prime}\left( {\nu^{\prime}} \right) + \left( {1 - p^{\prime}} \right)X^{\prime}\left( {\nu^{\prime\prime}} \right)} \right) > 0$.

### Proof of Corollary 2.2

#### Proof for $\Delta {\text{EVL}}$

Since $L_{{{\text{EVL}}}}^{*} < 0$, $\exists \delta^{\prime} < \delta^{*}$, such that $\Delta {\text{EVL}}\left( {Y,X\left( {\delta^{\prime}} \right)} \right) < 0$. Moreover, since $\Delta {\text{EVL}}\left( {Y,X\left( \delta \right)} \right)$ is linearly decreasing in $\delta$ for $\delta > \delta^{*}$, $\exists \delta^{\prime\prime} > \delta^{*} ,$ such that $\Delta {\text{EVL}}\left( {Y,X\left( {\delta^{\prime\prime}} \right)} \right) < 0$. Moreover, since $R_{{{\text{EVL}}}}^{*} > 0$, $\Delta {\text{EVL}}$ is continuous for $\delta \in \left( {\delta^{*} ,1} \right)$, and $\Delta {\text{EVL}}\left( {Y,X\left( {\delta^{\prime\prime}} \right)} \right) < 0$, the intermediate-value theorem implies that $\exists \gamma \in \left( {\delta^{*} ,\delta^{\prime\prime}} \right)$ such that $\Delta {\text{EVL}}\left( {Y,X\left( \gamma \right)} \right) > 0$. Since $\delta^{\prime} < \delta^{*}$, it follows that $\gamma \in \left( {\delta^{\prime},\delta^{\prime\prime}} \right)$. This implies that $\exists p \in \left( {0,1} \right)$, such that $\gamma = p\delta^{\prime} + \left( {1 - p} \right)\delta^{\prime\prime}$. As such, simple algebra confirms that $S_{X} \left( {1 - \gamma } \right) = pS_{X} \left( {1 - \delta^{\prime}} \right) + \left( {1 - p} \right)S_{X} \left( {1 - \delta^{\prime\prime}} \right)$.^12^ Therefore, since $X\left( \gamma \right)$, $X\left( {\delta^{\prime}} \right)$, and $X\left( {\delta^{\prime\prime}} \right)$ all share the same quality-of-life profiles, it follows further that $X\left( \gamma \right)$ produces the same survival and quality-of-life profile as $pX\left( {\delta^{\prime}} \right) + \left( {1 - p} \right)X\left( {\delta^{\prime\prime}} \right)$. Therefore, since $\Delta {\text{EVL}}\left( {Y,X\left( \gamma \right)} \right) > 0$, $\Delta {\text{EVL}}\left( {Y,pX\left( {\delta^{\prime}} \right) + \left( {1 - p} \right)X\left( {\delta^{\prime\prime}} \right)} \right) > 0$. *QED.*

#### Proof for $\Delta {\text{HYT}}$

Since $L_{{{\text{HYT}}}}^{*} < 0$, $\exists \nu^{\prime} < \delta^{*}$, such that $\Delta {\text{HYT}}\left( {Y^{\prime},X^{\prime}\left( {\nu^{\prime}} \right)} \right) < 0$. Moreover, since $\Delta {\text{HYT}}\left( {Y^{\prime},X^{\prime}\left( \delta \right)} \right)$ is linearly decreasing in $\delta$ for $\delta > \delta^{*}$, $\exists \nu^{\prime\prime} > \delta^{*} ,$ such that $\Delta {\text{HYT}}\left( {Y^{\prime},X^{\prime}\left( {\nu^{\prime\prime}} \right)} \right) < 0$. Moreover, since $R_{{{\text{HYT}}}}^{*} > 0$, $\Delta {\text{HYT}}$ is continuous for $\delta \in \left( {\delta^{*} ,1} \right)$, and $\Delta {\text{HYT}}\left( {Y^{\prime},X^{\prime}\left( {\nu^{\prime\prime}} \right)} \right) < 0$, the intermediate-value theorem implies that $\exists \gamma ^{\prime} \in \left( {\delta^{*} ,\nu^{\prime\prime}} \right)$ such that $\Delta {\text{HYT}}\left( {Y^{\prime},X^{\prime}\left( {\gamma ^{\prime}} \right)} \right) > 0$. Since $\nu^{\prime} < \delta^{*}$, it follows that $\gamma ^{\prime} \in \left( {\nu^{\prime},\nu^{\prime\prime}} \right)$. This implies that $\exists p^{\prime} \in \left( {0,1} \right)$, such that $\gamma ^{\prime} = p^{\prime}\nu^{\prime} + \left( {1 - p^{\prime}} \right)\nu^{\prime\prime}$. As such, simple algebra confirms that $S_{{X^{\prime}}} \left( {1 - \gamma ^{\prime}} \right) = p^{\prime}S_{{X^{\prime}}} \left( {1 - \nu^{\prime}} \right) + \left( {1 - p^{\prime}} \right)S_{{X^{\prime}}} \left( {1 - \nu^{\prime\prime}} \right)$.^13^ Therefore, since $X^{\prime}\left( {\gamma ^{\prime}} \right)$, $X^{\prime}\left( {\nu^{\prime}} \right)$, and $X^{\prime}\left( {\nu^{\prime\prime}} \right)$ all share the same quality-of-life profiles, it follows further that $X^{\prime}\left( {\gamma ^{\prime}} \right)$ produces the same survival and quality-of-life profile as $p^{\prime}X^{\prime}\left( {\nu^{\prime}} \right) + \left( {1 - X^{\prime}} \right)Y^{\prime}\left( {\nu^{\prime\prime}} \right)$. Therefore, since $\Delta {\text{HYT}}\left( {Y^{\prime},X^{\prime}\left( {\gamma^{\prime}} \right)} \right) > 0$, it follows that $\Delta {\text{HYT}}\left( {Y,p^{\prime}X^{\prime}\left( {\nu^{\prime}} \right) + \left( {1 - p^{\prime}} \right)X^{\prime}\left( {\nu^{\prime\prime}} \right)} \right) > 0$. *QED.*

##### Theorem 3

(Unbounded value of a life-year) *Suppose that* $Y$ *is a novel intervention with survival and quality-of-life profiles* $\left\{ {S_{Yt} ;Q_{Yt} } \right\}_{t = 1}^{T}$, *and* $X\left( \alpha \right)$ *is a family of standard-of-care interventions with profiles* $\left\{ {S_{Xt} \alpha ;Q_{Xt} } \right\}_{t = 1}^{T}$. *The terms*, $\delta^{*}$, $\underline {B}_{{{\text{EVL}}}}$, $\overline{B}_{{{\text{EVL}}}}$, $\underline {B}_{{{\text{HYT}}}}$, and $\overline{B}_{{{\text{HYT}}}}$ *are as defined in the text*. $\Delta {\text{EVL}}$ and $\Delta {\text{HYT}}$ *violate Principle* (3), *in the following sense*. Suppose $\sum\nolimits_{t} {\left( {S_{Yt} - S_{Xt} \delta^{*} } \right)Q_{Yt} < 0}$, so that $R_{{{\text{EVL}}}}^{*} < L_{{{\text{EVL}}}}^{*}$. For any $V > 0$, $\exists \delta^{\prime} \in \left( {0,\delta^{*} } \right)$ and $\delta^{\prime\prime} \in \left( {\delta^{*} ,1} \right)$, such that $\frac{{\left( {\Delta {\text{EVL}}\left( {Y,X\left( {\delta^{\prime}} \right)} \right) - \Delta {\text{EVL}}\left( {Y,X\left( {\delta^{\prime\prime}} \right)} \right)} \right)}}{{\mathop \sum \nolimits_{t} S_{{X\left( {\delta ^{\prime\prime}} \right)t}} - \mathop \sum \nolimits_{t} S_{{X\left( {\delta ^{\prime}} \right)t}} }} > V$. In contrast, suppose $\sum\nolimits_{t} {\left( {S_{Yt} - S_{Xt} \delta^{*} } \right)Q_{Xt} < 0}$, so that $R_{{{\text{HYT}}}}^{*} < L_{{{\text{HYT}}}}^{*}$. For any $V > 0$, $\exists \delta^{\prime} \in \left( {0,\delta^{*} } \right)$ and $\delta^{\prime\prime} \in \left( {\delta^{*} ,1} \right)$, such that $\frac{{\left( {\Delta {\text{HYT}}\left( {Y,X\left( {\delta^{\prime}} \right)} \right) - \Delta {\text{HYT}}\left( {Y,X\left( {\delta^{\prime\prime}} \right)} \right)} \right)}}{{\mathop \sum \nolimits_{t} S_{{X\left( {\delta ^{\prime\prime}} \right)t}} - \mathop \sum \nolimits_{t} S_{{X\left( {\delta ^{\prime}} \right)t}} }} > V$.

### Proof of Theorem 3

#### Proof for $\Delta {\text{EVL}}$

Pick some positive number, $V > 0$. We need to show that $\exists \delta^{\prime} \in \left( {0,\delta^{*} } \right)$ and $\delta^{\prime\prime} \in \left( {\delta^{*} ,1} \right)$, such that $\frac{{\left( {\Delta {\text{EVL}}\left( {Y,X\left( {\delta^{\prime}} \right)} \right) - \Delta {\text{EVL}}\left( {Y,X\left( {\delta^{\prime\prime}} \right)} \right)} \right)}}{{\mathop \sum \nolimits_{t} S_{{X\left( {\delta^{\prime\prime}} \right)t}} - \mathop \sum \nolimits_{t} S_{{X\left( {\delta^{\prime}} \right)t}} }} > V$.

Since $\Delta {\text{EVL}}$ is continuous and monotonic decreasing on either side of $\delta^{*}$, there must exist ${{\Delta^{\prime}}} < \delta^{*}$ and ${{\Delta^{\prime\prime}}} > \delta^{*} ,$ such that $\left( {\Delta {\text{EVL}}\left( {Y,X\left( {\Delta^{\prime}} \right)} \right) - \Delta {\text{EVL}}\left( {Y,X\left( {\Delta^{\prime\prime}} \right)} \right)} \right) > L_{{{\text{EVL}}}}^{*} - R_{{{\text{EVL}}}}^{*} > 0$. If $\frac{{L_{{{\text{EVL}}}}^{*} - R_{{{\text{EVL}}}}^{*} }}{{\mathop \sum \nolimits_{t} S_{{X\left( {\Delta^{\prime\prime}} \right)t}} - \mathop \sum \nolimits_{t} S_{{X\left( {\Delta^{\prime}} \right)t}} }} > V$, then $\frac{{\Delta {\text{EVL}}\left( {Y,X\left( {\Delta^{\prime}} \right)} \right) - \Delta {\text{EVL}}\left( {Y,X\left( {\Delta^{\prime\prime}} \right)} \right)}}{{\mathop \sum \nolimits_{t} S_{{X\left( {\Delta^{\prime\prime}} \right)t}} - \mathop \sum \nolimits_{t} S_{{X\left( {\Delta^{\prime}} \right)t}} }} > V$, and the proof is complete.

Therefore, it suffices to restrict our attention to the alternate case, in which $\frac{{L_{{{\text{EVL}}}}^{*} - R_{{{\text{EVL}}}}^{*} }}{{\mathop \sum \nolimits_{t} S_{{X\left( {\Delta^{\prime\prime}} \right)t}} - \mathop \sum \nolimits_{t} S_{{X\left( {\Delta^{\prime}} \right)t}} }} \le V$. In light of this supposition, and since $\sum\nolimits_{t} {S_{{X\left( {\Delta^{\prime\prime}} \right)t}} } - \sum\nolimits_{t} {S_{{X\left( {\Delta^{\prime}} \right)t}} } = \left( {{{\Delta^{\prime\prime}}} - {{\Delta^{\prime}}}} \right)\sum\nolimits_{t} {S_{Xt} }$, $\exists n > 1$, such that $\left( {1 - \frac{1}{n}} \right) = \frac{{L_{{{\text{EVL}}}}^{*} - R_{{{\text{EVL}}}}^{*} }}{{2V\left( {{{\Delta^{\prime\prime}}} - {{\Delta^{\prime}}}} \right)\mathop \sum \nolimits_{t} S_{Xt} }}$.

Now, since ${{\Delta^{\prime\prime}}} > \delta^{*}$, we can select $\delta^{\prime\prime} = {{\Delta^{\prime\prime}}} - \left( {{{\Delta^{\prime\prime}}} - \delta^{*} } \right)\left( \frac{1}{n} \right) > \delta^{*}$. Since ${{\Delta^{\prime}}} < \delta^{*}$, we can also select $\delta^{\prime} = {{\Delta^{\prime}}} + \left( {\delta^{*} - {{\Delta^{\prime}}}} \right)\left( \frac{1}{n} \right) < \delta^{*}$. Simple algebra reveals that $\delta^{\prime\prime} - \delta^{\prime} = {{\Delta^{\prime\prime}}} - \left( {{{\Delta^{\prime\prime}}} - {\updelta }^{*} } \right)\left( \frac{1}{n} \right) - {{\Delta^{\prime}}} - \left( {\delta^{*} - {{\Delta^{\prime}}}} \right)\left( \frac{1}{n} \right) = {{\Delta^{\prime\prime}}} - {{\Delta^{\prime}}} + \left( \frac{1}{n} \right)\left( { - \left( {{{\Delta^{\prime\prime}}} - {\updelta }^{*} } \right) - \left( {\delta^{*} - {{\Delta^{\prime}}}} \right)} \right) = \left( {{{\Delta^{\prime\prime}}} - {{\Delta^{\prime}}}} \right)\left( {1 - \frac{1}{n}} \right)$. Therefore, it follows that:

46 $$\frac{{L_{{{\text{EVL}}}}^{*} - R_{{{\text{EVL}}}}^{*} }}{{\left( {\delta^{\prime\prime} - \delta^{\prime}} \right)\mathop \sum \nolimits_{t} S_{Xt} }} = \frac{{L_{{{\text{EVL}}}}^{*} - R_{{{\text{EVL}}}}^{*} }}{{\left( {\Delta^{\prime\prime} - \Delta^{\prime}} \right)\mathop \sum \nolimits_{t} S_{Xt} \left( {1 - \frac{1}{n}} \right)}} = 2V.$$

Since ${{\Delta^{\prime}}} < \delta^{\prime} < \delta^{*}$ and ${{\Delta^{\prime\prime}}} > \delta^{\prime\prime} > \delta^{*}$, it follows that $\Delta{\text{EVL}}\left( {Y,X\left( {\delta^{\prime}} \right)} \right) - \Delta{\text{EVL}}\left( {Y,X\left( {\delta^{\prime\prime}} \right)} \right) > L_{{{\text{EVL}}}}^{*} - R_{{{\text{EVL}}}}^{*}$, so that $\frac{{L_{{{\text{EVL}}}}^{*} - R_{{{\text{EVL}}}}^{*} }}{{\left( {\delta^{\prime\prime} - \delta^{\prime}} \right)\mathop \sum \nolimits_{t} S_{Xt} }} < \frac{{\Delta {\text{EVL}}\left( {Y,X\left( {\delta^{\prime}} \right)} \right) - \Delta {\text{EVL}}\left( {Y,X\left( {\delta^{\prime\prime}} \right)} \right)}}{{\left( {\delta^{\prime\prime} - \delta^{\prime}} \right)\mathop \sum \nolimits_{t} S_{Xt} }}$. It then follows that $\frac{{\Delta {\text{EVL}}\left( {Y,X\left( {\delta^{\prime}} \right)} \right) - \Delta {\text{EVL}}\left( {Y,X\left( {\delta^{\prime\prime}} \right)} \right)}}{{\left( {\delta^{\prime\prime} - \delta^{\prime}} \right)\mathop \sum \nolimits_{t} S_{Xt} }} > 2V$.

Since $\left( {\delta^{\prime\prime} - \delta^{\prime}} \right)\sum\nolimits_{t} {S_{Xt} } = \sum\nolimits_{t} {S_{{X\left( {\delta^{\prime\prime}} \right)t}} } - \sum\nolimits_{t} {S_{{X\left( {\delta^{\prime}} \right)t}} }$, the theorem is proven. *QED.*

#### Proof for $\Delta {\text{HYT}}$

Pick some positive number, $V > 0$. We need to show that $\exists \delta^{\prime} \in \left( {0,\delta^{*} } \right)$ and $\delta^{\prime\prime} \in \left( {\delta^{*} ,1} \right)$, such that $\frac{{\left( {\Delta {\text{HYT}}\left( {Y,X\left( {\delta^{\prime}} \right)} \right) - \Delta {\text{HYT}}\left( {Y,X\left( {\delta^{\prime\prime}} \right)} \right)} \right)}}{{\mathop \sum \nolimits_{t} S_{{X\left( {\delta^{\prime\prime}} \right)t}} - \mathop \sum \nolimits_{t} S_{{X\left( {\delta^{\prime}} \right)t}} }} > V$.

Since $\Delta {\text{HYT}}$ is continuous and monotonic decreasing on either side of $\delta^{*}$, there must exist ${{\Delta^{\prime}}} < \delta^{*}$ and ${{\Delta^{\prime\prime}}} > \delta^{*} ,$ such that $\left( {\Delta {\text{HYT}}\left( {Y,X\left( {\Delta^{\prime}} \right)} \right) - \Delta {\text{HYT}}\left( {Y,X\left( {\Delta^{\prime\prime}} \right)} \right)} \right) > L_{{{\text{HYT}}}}^{*} - R_{{{\text{HYT}}}}^{*} > 0$. If $\frac{{L_{{{\text{HYT}}}}^{*} - R_{{{\text{HYT}}}}^{*} }}{{\mathop \sum \nolimits_{t} S_{{X\left( {\Delta^{\prime\prime}} \right)t}} - \mathop \sum \nolimits_{t} S_{{X\left( {\Delta^{\prime}} \right)t}} }} > V$, then $\frac{{\Delta{\text{HYT}}\left( {Y,X\left( {\Delta^{\prime}} \right)} \right) - \Delta{\text{HYT}}\left( {Y,X\left( {\Delta^{\prime\prime}} \right)} \right)}}{{\mathop \sum \nolimits_{t} S_{{X\left( {\Delta^{\prime\prime}} \right)t}} - \mathop \sum \nolimits_{t} S_{{X\left( {\Delta^{\prime}} \right)t}} }} > V$, and the proof is complete.

Therefore, it suffices to restrict our attention to the alternate case, in which $\frac{{L_{{{\text{HYT}}}}^{*} - R_{{{\text{HYT}}}}^{*} }}{{\mathop \sum \nolimits_{t} S_{{X\left( {\Delta^{\prime\prime}} \right)t}} - \mathop \sum \nolimits_{t} S_{{X\left( {\Delta^{\prime}} \right)t}} }} \le V$. In light of this supposition, and since $\sum\nolimits_{t} {S_{{X\left( {\Delta^{\prime\prime}} \right)t}} } - \sum\nolimits_{t} {S_{{X\left( {\Delta^{\prime}} \right)t}} } = \left( {{{\Delta^{\prime\prime}}} - {{\Delta^{\prime}}}} \right)\sum\nolimits_{t} {S_{Xt} }$, $\exists n > 1$, such that $\left( {1 - \frac{1}{n}} \right) = \frac{{L_{{{\text{HYT}}}}^{*} - R_{{{\text{HYT}}}}^{*} }}{{2V\left( {{{\Delta^{\prime\prime}}} - {{\Delta^{\prime}}}} \right)\mathop \sum \nolimits_{t} S_{Xt} }}$.

Now, since ${{\Delta^{\prime\prime}}} > \delta^{*}$, we can select $\delta^{\prime\prime} = {{\Delta^{\prime\prime}}} - \left( {{{\Delta^{\prime\prime}}} - \delta^{*} } \right)\left( \frac{1}{n} \right) > \delta^{*}$. Since ${{\Delta^{\prime}}} < \delta^{*}$, we can also select $\delta^{\prime} = {{\Delta^{\prime}}} + \left( {\delta^{*} - {{\Delta^{\prime}}}} \right)\left( \frac{1}{n} \right) < \delta^{*}$. Simple algebra reveals that $\delta^{\prime\prime} - \delta^{\prime} = {{\Delta^{\prime\prime}}} - \left( {{{\Delta^{\prime\prime}}} - {\updelta }^{*} } \right)\left( \frac{1}{n} \right) - {{\Delta^{\prime}}} - \left( {\delta^{*} - {{\Delta^{\prime}}}} \right)\left( \frac{1}{n} \right) = {{\Delta^{\prime\prime}}} - {{\Delta^{\prime}}} + \left( \frac{1}{n} \right)\left( { - \left( {{{\Delta^{\prime\prime}}} - {\updelta }^{*} } \right) - \left( {\delta^{*} - {{\Delta^{\prime}}}} \right)} \right) = \left( {{{\Delta^{\prime\prime}}} - {{\Delta^{\prime}}}} \right)\left( {1 - \frac{1}{n}} \right)$. Therefore, it follows that:

47 $$\frac{{L_{{{\text{HYT}}}}^{*} - R_{{{\text{HYT}}}}^{*} }}{{\left( {\delta^{\prime\prime} - \delta^{\prime}} \right)\mathop \sum \nolimits_{t} S_{Xt} }} = \frac{{L_{{{\text{HYT}}}}^{*} - R_{{{\text{HYT}}}}^{*} }}{{\left( {\Delta^{\prime\prime} - \Delta^{\prime}} \right)\mathop \sum \nolimits_{t} S_{Xt} \left( {1 - \frac{1}{n}} \right)}} = 2V.$$

Since ${{\Delta^{\prime}}} < \delta^{\prime} < \delta^{*}$ and ${{\Delta^{\prime\prime}}} > \delta^{\prime\prime} > \delta^{*}$, it follows that $\Delta {\text{HYT}}\left( {Y,X\left( {\delta^{\prime}} \right)} \right) - \Delta {\text{HYT}}\left( {Y,X\left( {\delta^{\prime\prime}} \right)} \right) > L_{{{\text{HYT}}}}^{*} - R_{{{\text{HYT}}}}^{*}$, so that $\frac{{L_{{{\text{HYT}}}}^{*} - R_{{{\text{HYT}}}}^{*} }}{{\left( {\delta^{\prime\prime} - \delta^{\prime}} \right)\mathop \sum \nolimits_{t} S_{Xt} }} < \frac{{\Delta {\text{HYT}}\left( {Y,X\left( {\delta^{\prime}} \right)} \right) - \Delta {\text{HYT}}\left( {Y,X\left( {\delta^{\prime\prime}} \right)} \right)}}{{\left( {\delta^{\prime\prime} - \delta^{\prime}} \right)\mathop \sum \nolimits_{t} S_{Xt} }}$. It then follows that $\frac{{\Delta {\text{HYT}}\left( {Y,X\left( {\delta^{\prime}} \right)} \right) - \Delta {\text{HYT}}\left( {Y,X\left( {\delta^{\prime\prime}} \right)} \right)}}{{\left( {\delta^{\prime\prime} - \delta^{\prime}} \right)\mathop \sum \nolimits_{t} S_{Xt} }} > 2V$.

Since $\left( {\delta^{\prime\prime} - \delta^{\prime}} \right)\sum\nolimits_{t} {S_{Xt} } = \sum\nolimits_{t} {S_{{X\left( {\delta^{\prime\prime}} \right)t}} } - \sum\nolimits_{t} {S_{{X\left( {\delta^{\prime}} \right)t}} }$, the theorem is proven. *QED.*

##### Theorem 4:

(The need for discontinuity in EVL and HYT) *Suppose there are two interventions*, *A* and *B*, with sequences of quality-of-life and survival, $\left\{ {S_{At} ;Q_{At} } \right\}_{t = 0}^{t = T}$ and $\left\{ {S_{Bt} ;Q_{Bt} } \right\}_{t = 0}^{t = T}$. And suppose $\Delta {\text{Metric}}^{\prime}$ and $\Delta {\text{Metric}}^{\prime\prime}$ are as defined in Eqs. (17) and (18), respectively. Suppose that the following two conditions hold:

48 $$\mathop \sum \limits_{t} S_{Bt} \le \mathop \sum \limits_{t} S_{At}$$

49 $$\mathop \sum \limits_{t} \left( {S_{Bt} } \right)\left( {Q_{At} - Q_{Bt} } \right) > \mathop \sum \limits_{t} \left( {S_{At} } \right)\left( {Q_{At} - Q_{Bt} } \right) > 0.$$

Under these circumstances, if $\Delta {\text{Metric}}^{\prime}\left( {B,A} \right) > 0,$ then $\Delta {\text{Metric}}^{\prime}\left( {A,B} \right) > 0$, and if $\Delta {\text{Metric}}^{\prime\prime}\left( {B,A} \right) > 0$, then $\Delta {\text{Metric}}^{\prime\prime}\left( {A,B} \right) > 0$.

### Proof of Theorem 4

Suppose $\Delta {\text{Metric}}^{\prime}\left( {B,A} \right) > 0$. Inequality (48) implies

50 $$\mathop \sum \limits_{t} \left( {S_{At} - S_{Bt} } \right) \ge 0 \ge \mathop \sum \limits_{t} \left( {S_{Bt} - S_{At} } \right).$$

The left-hand inequality in (49) implies

51 $$\mathop \sum \limits_{t} S_{Bt} \left( {Q_{At} - Q_{Bt} } \right) > \mathop \sum \limits_{{\text{t}}} S_{At} \left( {Q_{At} - Q_{Bt} } \right) > 0 > \mathop \sum \limits_{{\text{t}}} S_{At} \left( {Q_{Bt} - Q_{At} } \right).$$

The first inequality in this expression follows from the left-hand inequality in (49) and the second inequality follows from the right-hand inequality in (49). Adding these two conditions together implies

52 $$\mathop \sum \limits_{t} \left( {S_{At} - S_{Bt} } \right) + \mathop \sum \limits_{t} S_{Bt} \left( {Q_{At} - Q_{Bt} } \right) > \mathop \sum \limits_{t} \left( {S_{Bt} - S_{At} } \right) + \mathop \sum \limits_{{\text{t}}} S_{At} \left( {Q_{Bt} - Q_{At} } \right).$$

This then proves the desired result

53 $$\Delta {\text{Metric}}^{\prime}\left( {A,B} \right) > \Delta {\text{Metric}}^{\prime}\left( {B,A} \right) > 0.$$

Now suppose $\Delta {\text{Metric}}^{\prime\prime}\left( {B,A} \right) > 0$. Since $\Delta {\text{Metric}}^{\prime}\left( {A,B} \right) > \Delta {\text{Metric}}^{\prime}\left( {B,A} \right)$, it follows that:

$$\Delta {\text{Metric}}^{\prime}\left( {A,B} \right) + \mathop \sum \limits_{t} \left( {S_{At} - S_{Bt} } \right)\left( {Q_{At} - Q_{Bt} } \right) > \Delta {\text{Metric}}^{\prime}\left( {B,A} \right) + \mathop \sum \limits_{t} \left( {S_{Bt} - S_{At} } \right)\left( {Q_{Bt} - Q_{At} } \right).$$

Simple algebra reveals that the left-hand side of the inequality is equal to $\Delta {\text{Metric}}^{\prime\prime}\left( {A,B} \right)$ and the right-hand side is equal to $\Delta {\text{Metric}}^{\prime\prime}\left( {B,A} \right)$.^14^ The desired result then follows.

It remains to check that the theorem’s conditions can be met. Consider a two-period setting, where $\left\{ {S_{At} } \right\} = \left\{ {0.6,0.6} \right\}$, $\left\{ {S_{Bt} } \right\} = \left\{ {0.7,0.5} \right\}$, and $\left\{ {Q_{Bt} - Q_{At} } \right\} = \left\{ {0.9,0.5} \right\}$. It is simple to verify that inequalities (48) and (49) are met in this example. *QED.*

#### Theorem 5

(Principle-consistency of GRACE and QALY metrics) *Define the metrics*, $\Delta {\text{QALY}}$ *and* $\Delta {\text{GRACE}}$ *as specified in Eqs*. (1) and (4), *respectively. Let* $W$ *be any continuously differentiable and weakly monotonic HRQoL utility function defined over the domain*, $Q \in \left[ {0,1} \right]$, *where* $W\left( Q \right) \ge 0$ *and* $W\left( 0 \right) = 0$. *For a given pre-existing disability level bounded away from zero in the sense that* $\exists \underline {d} \in \left( {0,1} \right)$, *such that* $d^{*} \ge \underline {d} > 0$, *both* $\Delta {\text{QALY}}$ *and* $\Delta {\text{GRACE}}$ *satisfy Principles* (1), (1), (2), (3), *and* (4).

### Proof of Theorem 5

Since $\Delta {\text{QALY}}$ is the special case of $\Delta {\text{GRACE}}$ that arises when $W\left( Q \right) = Q$, it suffices to prove that $\Delta {\text{GRACE}}$ is consistent with the listed Principles.

An equivalent way of rewriting the definition of $\Delta {\text{GRACE}}$ in Eq. (4) is

54 $$\Delta {\text{GRACE}}\left( {Y,X;Q_{0} } \right) = \mathop \sum \limits_{t} \left[ {S_{Yt} \frac{{W\left( {Q_{Yt} } \right)}}{{W\left( {Q_{0} } \right)}} - S_{Xt} \frac{{W\left( {Q_{Xt} } \right)}}{{W\left( {Q_{0} } \right)}}} \right].$$

This formulation makes it clear that $\Delta {\text{GRACE}}\left( {X^{\prime},X;Q_{0} } \right) = \Delta {\text{GRACE}}\left( {Y,X;Q_{0} } \right) - \Delta {\text{GRACE}}\left( {Y,X^{\prime};Q_{0} } \right)$. This eases the proof of Principle (1). Suppose there exist technologies $X^{\prime}$ and $X$, such that $\Delta {\text{GRACE}}\left( {X^{\prime},X;Q_{0} } \right) > 0$ for a given $Q_{0}$. Since $\Delta {\text{GRACE}}\left( {X^{\prime},X;Q_{0} } \right) = \Delta {\text{GRACE}}\left( {Y,X;Q_{0} } \right) - \Delta {\text{GRACE}}\left( {Y,X^{\prime};Q_{0} } \right)$, it follows that $\Delta {\text{GRACE}}\left( {Y,X;Q_{0} } \right) - \Delta {\text{GRACE}}\left( {Y,X^{\prime};Q_{0} } \right) > 0$. Moving in the other direction, suppose $\Delta {\text{GRACE}}\left( {Y,X;Q_{0} } \right) - \Delta {\text{GRACE}}\left( {Y,X^{\prime};Q_{0} } \right) > 0$. It then follows that $\Delta {\text{GRACE}}\left( {X^{\prime},X;Q_{0} } \right) = \Delta {\text{GRACE}}\left( {Y,X;Q_{0} } \right) - \Delta {\text{GRACE}}\left( {Y,X^{\prime};Q_{0} } \right) > 0$.

Turning to Condition (1), suppose there exist interventions $A$, $B$, $C$, and $D$ such that

55 $$\mathop \sum \limits_{t} S_{At} \left( {\frac{{W(Q_{At} )}}{{W\left( {Q_{0} } \right)}}} \right) - \mathop \sum \limits_{t} S_{Bt} \left( {\frac{{W(Q_{Bt} )}}{{W\left( {Q_{0} } \right)}}} \right) > \mathop \sum \limits_{t} S_{Ct} \left( {\frac{{W(Q_{Ct} )}}{{W\left( {Q_{0} } \right)}}} \right) - \mathop \sum \limits_{t} S_{Dt} \left( {\frac{{W(Q_{Dt} )}}{{W\left( {Q_{0} } \right)}}} \right),$$

which upon algebraic manipulation gives the desired result

56 $$\mathop \sum \limits_{t} S_{At} \left( {\frac{{W(Q_{At} )}}{{W\left( {Q_{0} } \right)}}} \right) - \mathop \sum \limits_{t} S_{Ct} \left( {\frac{{W(Q_{Ct} )}}{{W\left( {Q_{0} } \right)}}} \right) > \mathop \sum \limits_{t} S_{Bt} \left( {\frac{{W(Q_{Bt} )}}{{W\left( {Q_{0} } \right)}}} \right) - \mathop \sum \limits_{t} S_{Dt} \left( {\frac{{W(Q_{Dt} )}}{{W\left( {Q_{0} } \right)}}} \right).$$

Turning to Principle (2), suppose that interventions $X$ and $Y$ produce identical quality-of-life, $\left\{ {Q_{t} } \right\}_{t = 1}^{T}$, $S_{Yt} \ge S_{Xt}$ $\forall t$, and $S_{Yt} > S_{Xt}$ for some $t$. Since $W\left( Q \right) \ge 0, \forall Q$, it follows that $\Delta {\text{GRACE}}\left( {Y,X;Q_{0} } \right) > 0$.

Turning to Principle (3), ${\Delta }GRACE$ is continuously differentiable for all nonnegative survival sequences. Therefore, $\frac{{{\text{d}}\Delta {\text{GRACE}}\left( {Y,X;d^{*} } \right)}}{{{\text{d}}S_{Xt} }} = - \frac{{W\left( {Q_{Xt} } \right)}}{{W\left( {Q_{0} } \right)}}$. Since $W$ is weakly monotonic, $0 > \frac{{{\text{d}}\Delta {\text{GRACE}}\left( {Y,X;Q_{0} } \right)}}{{{\text{d}}S_{Xt} }} \ge - \frac{W\left( 1 \right)}{{W\left( {1 - \underline{{Q_{0} }} } \right)}}$. Therefore, for any pair of technologies $\left( {L,L^{\prime}} \right)$ with bounded difference in survival, $\frac{{\left( {\Delta {\text{GRACE}}\left( {Y,L} \right) - \Delta {\text{GRACE}}\left( {Y,L^{\prime}} \right)} \right)}}{{\mathop \sum \nolimits_{t} S_{{L^{\prime}t}} - \mathop \sum \nolimits_{t} S_{Lt} }} = - \frac{{{\text{d}}\Delta {\text{GRACE}}\left( {Y,X;Q_{0} } \right)}}{{{\text{d}}S_{Xt} }}\left( {\mathop \sum \limits_{t} S_{{L^{\prime}t}} - \mathop \sum \limits_{t} S_{Lt} } \right) < \lambda \frac{W\left( 1 \right)}{{W\left( {1 - \underline{{Q_{0} }} } \right)}}$.

To prove consistency with Principle (4), consider technologies, $X$, $Y$, and $Z$, such that $\Delta {\text{GRACE}}\left( {Z,X;Q_{0} } \right) > 0$, $\Delta {\text{GRACE}}\left( {Z,Y;Q_{0} } \right) > 0$, and $X$ and $Y$ produce equal quality-of-life. Pick some $0 < p < 1$, and define $pX + \left( {1 - p} \right)Y$ as the intervention that uses $X$ on the fraction, $p$, of the population, and $Y$ on the fraction, $1 - p$, of the population. Also pick some baseline pre-illness health level $Q_{0}$. Since $\Delta {\text{GRACE}}\left( {Z,X;Q_{0} } \right) > 0$ and $\Delta {\text{GRACE}}\left( {Z,Y;Q_{0} } \right) > 0$, it follows that:

57 $$\mathop \sum \limits_{t} \left[ {S_{Zt} \frac{{W\left( {Q_{Zt} } \right)}}{{W\left( {Q_{0} } \right)}}} \right] > \mathop \sum \limits_{t} \left[ {S_{Xt} \frac{{W\left( {Q_{Xt} } \right)}}{{W\left( {Q_{0} } \right)}}} \right]$$

58 $$\mathop \sum \limits_{t} \left[ {S_{Zt} \frac{{W\left( {Q_{Zt} } \right)}}{{W\left( {Q_{0} } \right)}}} \right] > \mathop \sum \limits_{t} \left[ {S_{Yt} \frac{{W\left( {Q_{Yt} } \right)}}{{W\left( {Q_{0} } \right)}}} \right]$$

59 $$p\mathop \sum \limits_{t} \left[ {S_{Xt} \frac{{W\left( {Q_{Xt} } \right)}}{{W\left( {{\text{Q}}_{0} } \right)}}} \right] + \left( {1 - p} \right)\mathop \sum \limits_{t} \left[ {S_{Yt} \frac{{W\left( {Q_{Yt} } \right)}}{{W\left( {{\text{Q}}_{0} } \right)}}} \right] > \mathop \sum \limits_{t} \left[ {{\text{S}}_{{{\text{Zt}}}} \frac{{W\left( {Q_{Zt} } \right)}}{{W\left( {{\text{Q}}_{0} } \right)}}} \right].$$

The result follows, and this completes the proof of the theorem. *QED.*

#### Theorem 6

(Non-discrimination conditions for GRACE) *If utility over HRQoL*, $W\left( Q \right)$, *exhibits constant relative risk-aversion, and if* $\left( {1 - t_{j}^{*} } \right) = \left( {1 - d_{j}^{*} } \right)\left( {1 - t^{*} } \right)$ *for all Type* $j$ *patients, then GRACE complies with Principle* (5), *equity for the disabled and sick*.

### Proof of Theorem 6

We begin with a standard functional equation that is implied by the constant relative risk aversion condition: there exists some monotonic function, $g$, such that $W\left( {\alpha Q} \right) = g\left( \alpha \right) \cdot W\left( Q \right)$ for all $0 < \alpha \le 1$ [31, Theorem 3.1.1.4]. This implies the following chain of reasoning:

60 $$\begin{gathered} \frac{{W\left( {\left( {1 - {\text{t}}_{{\text{H}}}^{*} } \right){\text{Q}}_{{\text{P}}} } \right)}}{{W\left( {\left( {1 - {\text{d}}_{{\text{H}}}^{*} } \right){\text{Q}}_{{\text{P}}} } \right)}} = \frac{{W\left( {\left( {1 - d_{H}^{*} } \right)\left( {1 - t^{*} } \right){\text{Q}}_{{\text{P}}} } \right)}}{{W\left( {\left( {1 - d_{H}^{*} } \right)Q_{P} } \right)}} = \frac{{g\left( {1 - d_{H}^{*} } \right)W\left( {\left( {1 - t^{*} } \right)Q_{P} } \right)}}{{g\left( {1 - d_{H}^{*} } \right)W\left( {Q_{P} } \right)}} = \frac{{W\left( {\left( {1 - t^{*} } \right)Q_{P} } \right)}}{{W\left( {Q_{P} } \right)}} \hfill \\ \frac{{g\left( {\frac{1}{{1 - d_{L}^{*} }}} \right)W\left( {\left( {1 - d_{L}^{*} } \right)\left( {1 - t^{*} } \right)Q_{P} } \right)}}{{g\left( {\frac{1}{{1 - d_{L}^{*} }}} \right)W\left( {\left( {1 - d_{L}^{*} } \right)Q_{P} } \right)}} = \frac{{W\left( {\left( {1 - {\text{t}}_{{\text{L}}}^{*} } \right){\text{Q}}_{{\text{P}}} } \right)}}{{W\left( {\left( {1 - {\text{d}}_{{\text{L}}}^{*} } \right){\text{Q}}_{{\text{P}}} } \right)}} \hfill \\ \end{gathered}$$

Within expression (60), the first and last equalities follow from Theorem 6’s condition that $\left(1-{t}_{j}^{*}\right)=(1-{d}_{j}^{*})(1-{t}^{*})$; the second and fourth equalities follow from the functional equation result described above.

## Footnotes

1 Henceforth, when we denote ICER’s EVL metric simply as “EVL” or the “EVL metric.”.

2 As Campbell et al. explain [16], “When an intervention does not extend life, incremental evLYs and incremental QALYs are identical. Note that if more than one comparator exists, then the one with the fewest life-years (frequently ‘standard of care’) must be selected as the anchor comparator for all comparisons in order to estimate an intervention’s life extension and corresponding evLYs.”.

3 Some studies in the literature have documented stated preferences for immediate death over continued survival in low quality-of-life states [28, 29]. One can sharpen the principle here to apply specifically to quality-of-life states that are preferred to death, a typical assumption when defining an ordinal quality-of-life scale.

4 One might alternatively operationalize this concept as a bounded marginal value of survival gains. This approach would lead to qualitatively similar conclusions, as it would rule out value metrics that are anywhere discontinuous in total survival.

5 https://www.congress.gov/bill/117th-congress/house-bill/5376/text/rh.

6 If Principle (2) holds, it will always be true that $\Delta{\text{Metric}}\left( {Y,H} \right) \ge \Delta{\text{Metric}}\left( {Y,H^{\prime}} \right)$, but we do not require the validity of Principle (2) for this argument.

7 Here, we implicitly assume that any metric must satisfy $\Delta{\text{Metric}}\left( {Y,Y} \right) = 0$ for any technology, $Y$.

8 Here, we implicitly assume that any metric must satisfy $\Delta{\text{Metric}}\left( {A,A^{\prime}} \right) = - \Delta {\text{Metric}}\left( {A^{\prime},A} \right)$, for any pair of technologies, $A$ and $A^{\prime}$.

9 While the numbers would change, this anomaly would persist even if one were to use the “modified” formula when the standard-of-care produces more total survival than the novel technology. In this case, the survival profile used to compute expected quality-of-life gains would still change, allowing for the construction of anomalous examples like this.

10 The figure reflects the result that, on either side of the discontinuity, $\delta^{*}$, $\Delta {\text{EVL}}$ is continuously differentiable and linearly decreasing in the technology level, $\delta$.

11 Suitably modified proofs of these theorems are available on request from the authors.
